# Effect of Essential Phospholipids in Metabolic Dysfunction‐Associated Steatotic Liver Disease: A Randomised Phase 4 Clinical Trial

**DOI:** 10.1111/liv.70601

**Published:** 2026-03-26

**Authors:** Norbert Stefan, Marek Hartleb, Jiangao Fan, Münevver Demir, Jörn M. Schattenberg, Jan Gietka, Łukasz Bułdak, Branko Popovic, Rafael Varona, Beatrice Bois De Fer

**Affiliations:** ^1^ Department of Internal Medicine IV University Hospital Tübingen Tübingen Germany; ^2^ Department of Gastroenterology and Hepatology Medical University of Silesia Katowice Poland; ^3^ Department of Gastroenterology, Xinhua Hospital Shanghai Jiao Tong University School of Medicine Shanghai China; ^4^ Department of Hepatology and Gastroenterology, Charité Universitätsmedizin Berlin, Campus Virchow‐Klinikum and Campus Charité Mitte Berlin Germany; ^5^ Department of Internal Medicine II Saarland University Medical Center Homburg Germany; ^6^ PharmaScienceHub (PSH) Saarland University Saarbrücken Germany; ^7^ Hepatolodzy Outpatient Clinic Warsaw Poland; ^8^ Department of Internal Medicine and Clinical Pharmacology Medical University of Silesia Katowice Poland; ^9^ Opella Healthcare A. Nattermann & Cie. GmbH Frankfurt am Main Germany; ^10^ Opella Healthcare Group SAS Neuilly‐sur‐Seine France

**Keywords:** adjunctive therapy, fatigue, haemoglobin A1c (HbA1c), hepatic steatosis, quality of life

## Abstract

**Background and Aims:**

Metabolic dysfunction‐associated steatotic liver disease (MASLD) poses a significant health burden and impacts quality of life (QoL). This study evaluates the effects of essential phospholipids (EPL) on liver steatosis, QoL, and other liver and metabolic parameters in patients with MASLD and associated comorbidities.

**Methods:**

In this multicenter, double‐blind, randomised, placebo‐controlled phase 4 clinical trial, patients with MASLD and type 2 diabetes, hyperlipidemia, or obesity received either EPL or placebo, alongside standard of care. Primary endpoint: change in hepatic steatosis from baseline to 6 months (measured by Controlled Attenuation Parameter [CAP] score); secondary endpoints: changes in QoL (measured by the Chronic Liver Disease Questionnaire [CLDQ‐MASLD]), symptom changes; other endpoints: other liver, metabolic, and lipid parameters, and safety.

**Results:**

Of 193 randomised patients, 165 constituted the modified intention‐to‐treat population (median age: 56.5 years [EPL arm], 55.0 years [placebo arm]). More than ¾ of patients were obese and had CAP score ≥ 280 dB/m. At 6 months, EPL treatment significantly reduced CAP (*p* = 0.0269) versus placebo. This effect was evident at 3 months (*p* = 0.0049) and sustained until 3 months post treatment (*p* = 0.0234). QoL total score showed numerical improvement, with statistically significant improvement in fatigue subscore (*p* = 0.0229) with EPL versus placebo at 6 months. EPL significantly improved HbA1c levels (*p* = 0.0069) over 6 months. No safety concerns arose.

**Conclusions:**

The beneficial effects of EPL on hepatic steatosis, QoL and glycemic control, and its favourable safety profile make it a promising candidate for managing steatosis and enhancing overall liver health in MASLD patients with cardiometabolic risk.

**Trial Registration:**

The trial was registered in the EudraCT (2021‐006069‐39)

## Lay Summary

- Essential phospholipids (EPLs) can be used along with standard treatments to reduce liver fat in patients with fatty liver disease linked to metabolic health issues. However, strong evidence from well‐controlled studies is limited.
- The present phase 4 clinical study (EXCEL) found that EPLs, when added to standard care, significantly reduced liver fat, tiredness and blood sugar levels within 6 months in such patients.
- These results suggest that EPLs could be a safe and effective option to improve liver health in patients with fatty liver disease with conditions like diabetes, high cholesterol, or obesity.

**Abbreviations**
AEs: Adverse Events
CAP: Controlled Attenuation Parameter
CI: confidence interval
CLDQ: Chronic Liver Disease Questionnaire
EPL: essential phospholipids
HRQoL: health‐related quality of life
ITT: intention to treat
LS: least‐square
LSMD: least‐square mean difference
MASH: metabolic dysfunction‐associated steatohepatitis
MASLD: Metabolic dysfunction‐associated steatotic liver disease
mITT: modified intention to treat
PC: polyunsaturated phosphatidylcholine
PROs: patient‐reported outcomes
QoL: quality of life
SE: standard error
SoC: standard of care
TEAEs: treatment‐emergent AEs

## 1 Introduction

The global prevalence of metabolic dysfunction‐associated steatotic liver disease (MASLD) has been steadily rising over the past decades and is currently estimated at approximately 38% [1, 2]. The prevalence of MASLD is higher in people with obesity (75%) [3], type 2 diabetes (69%) [4], and dyslipidemia (20%–80%) [5]. Because MASLD is a growing medical problem in people with diabetes (particularly type 2 diabetes, especially when associated with obesity), most recently, a Consensus Report of the American Diabetes Association addressed the recent MASLD nomenclature change, risk stratification, current treatment and long‐term monitoring options and proposed an interprofessional approach to disease management in MASLD [6]. Furthermore, metabolic dysfunction‐associated steatohepatitis (MASH), a more severe form of MASLD that potentially progresses to advanced fibrosis, cirrhosis, and hepatocellular carcinoma, also poses a significant and escalating clinical and economic challenge worldwide [7, 8, 9]. Moreover, individuals with MASLD—particularly those affected by MASH and hepatic fibrosis—are at heightened risk of developing type 2 diabetes, cardiovascular disease, chronic kidney disease, and certain types of extrahepatic cancers [10, 11, 12]. Thus, MASLD may be regarded as another non‐communicable disease [13].

MASLD is not only associated with significant healthcare and socio‐economic burden, but it also markedly impacts the patients' health‐related quality of life (HRQoL), including fatigue, abdominal discomfort, and sleep disturbance [14, 15, 16]. The Chronic Liver Disease Questionnaire (CLDQ‐MASLD) is the only validated instrument to assess HRQoL in patients with MASLD and provides insights into patient‐reported outcomes (PROs) to help improve patient care [5, 14, 17, 18].

The widely accepted standard of care (SoC) for the first‐line management of MASLD is lifestyle intervention, including physical exercise and diet modification [9, 19, 20, 21]. Currently, resmetirom is a conditionally approved medication by the FDA as well as EMA for the treatment of noncirrhotic MASH in conjunction with diet and exercise [22, 23] and more recently, semaglutide has received FDA approval for the treatment of MASH in adults with moderate‐to‐advanced fibrosis [24]. This underscores the need for early intervention in steatosis to prevent progression to severe, irreversible stages, especially in high‐risk patients with metabolic burden. Early diagnosis and risk stratification of MASLD are critical to allow for early intervention and decision about the clinical care pathway. Non‐invasive tests like FibroScan are increasingly used due to their simplicity and safety for assessing steatosis by controlled attenuation parameter (CAP) and fibrosis by liver stiffness measures (LSM) [25]. A subset of MASLD patients develop MASH, placing them at the highest risk of progression to cirrhosis and adverse outcomes. Early identification of these patients with at‐risk MASH, defined by active steatohepatitis and advanced fibrosis (stage 2 or higher), is crucial [26, 27]. The FibroScan‐AST (FAST) score was developed as a non‐invasive tool to identify these patients who are at the highest risk of disease progression. It combines CAP and liver stiffness measurements from FibroScan with aspartate aminotransferase (AST) levels, allowing a single index to assess fibrosis and liver inflammation. It was subsequently validated in global studies indicating efficient non‐invasive identification of patients with at‐risk MASH [26, 27].

Among several pathophysiological mechanisms that promote MASLD, the phosphatidylcholine pathway is important. The levels of polyunsaturated phosphatidylcholine (PC) are lower in patients with MASLD, particularly MASH, compared to healthy individuals, affecting liver cell function [28, 29]. Essential phospholipids (EPL) or polyene phosphatidylcholine, derived from highly purified soybean extracts, contain over 72% of 3‐sn‐phosphatidyl with 1, 2‐dilinoleoylphosphatidylcholine as the main ingredient [30]. EPL has been evaluated in patients with MASLD across multiple clinical trials [30, 31]. While these studies were relatively small, collectively they provide supportive evidence for EPL as a beneficial adjunctive therapy in MASLD. In most trials, EPL, either as monotherapy or combined with other treatments, was associated with improvements in liver function markers, lipid profiles, and resolution or improvement of hepatic steatosis by ultrasonography [31]. A few studies showed that EPL treatment had some influence on the reduction of hepatic fibrosis in MASLD patients vs. control groups, as evidenced by improved Fibromax test results and transient elastography, with treated patients showing a trend for less advanced fibrosis stages [32, 33, 34]. However, larger multicenter trials are needed to further validate these anti‐fibrotic effects. With regard to the beneficial action of EPL at the cellular level, it exerts multiple effects—integrates into damaged cell membranes and helps stabilise and repair cell membranes, supports membrane‐dependent enzyme activities, enhances lipid metabolism and possesses antioxidant properties [35, 36, 37]. Through these actions EPL potentially attenuate steatosis and slow the progression of fibrosis.

The present study aimed to systematically assess the effects of EPL compared to placebo, on hepatic steatosis, QoL and safety, when added to SoC, in patients with MASLD who additionally have type 2 diabetes mellitus (T2DM), hyperlipidemia or obesity.

## 2 Methods

### 2.1 Study Design and Participants

This phase 4 clinical study, EXCEL (Exploring Efficacy of Essentiale in Fatty Liver Disease), was a multicenter, double‐blind, randomised, placebo‐controlled, parallel‐group trial conducted at 15 centers in Germany and Poland from November 2022 to May 2024. The trial included an enrolment visit, and two control visits at 3 and 6 months, and an end‐of‐study visit after a 3‐month post‐treatment follow‐up period (Figure S1). The detailed study design was published previously [38]. Briefly, the study enrolled patients (18 to 70 years of age) with MASLD, with a steatosis score of S1 to S3 (controlled attenuation parameter [CAP] score > 248 dB/m), a liver fibrosis score of F1 to F3 (defined by the Liver Stiffness Measurement [LSM] score of 5–13 kPa) [39, 40] as measured by FibroScan vibration‐controlled transient elastography (VCTE) and at least one comorbidity (T2DM, hyperlipidemia, or obesity). Eligible patients were randomised 1:1 to receive either EPL 1800 mg/day orally + SoC (EPL arm) or placebo + SoC (placebo arm). Herein, the SoC refers to lifestyle modifications, which included dietary adjustments (e.g., low‐to‐moderate fat and moderate‐to‐high carbohydrate intake, avoidance of fructose‐containing beverages and foods, and alcohol restriction) and increased physical exercise. Lifestyle modification (diet+exercise) was implemented as per the EASL‐EASD‐EASO 2016 Clinical Practice Guidelines for MASLD management [41] or site‐ and disease‐specific SoC [38]. The SoC practices by each patient were recorded in an electronic case report form [38] and their adherence was evaluated at 3 and 6 months. The S1 provides further details. All patients provided written informed consent, and the trial was approved by the Ethics Committee at the Medical Faculty of the Eberhard Karls University and University Hospital Tübingen, Germany.

### 2.2 Endpoints

The primary endpoint was the change in hepatic steatosis (measured by the CAP score [dB/m] obtained through Fibroscan VCTE) from baseline to 6 months in patients treated with EPL versus placebo. The secondary endpoints were changes from baseline to 6 months in QoL total score (measured by the CLDQ‐MASLD questionnaire, previously known as CLDQ‐NAFLD, and often mentioned in the literature and clinical practice as CLDQ NAFLD‐NASH), and symptoms evaluation for asthenia, feeling depressed, abdominal pain/discomfort, or fatigue (measured by the Global Overall Symptom [GOS] scale). Safety was assessed by the occurrence of any adverse events (AEs), including treatment‐emergent AEs (TEAEs), serious AEs (SAEs), and adverse events of special interest (AESIs).

The key exploratory endpoints included changes in QoL subscores, liver fibrosis (measured by LSM [kPa] obtained through Fibroscan VCTE), liver enzyme and blood lipid levels, evaluation for additional symptoms and patient satisfaction. More details on exploratory endpoints are provided in the Table S1. As a post hoc analysis, the changes in FAST score from baseline to 6 months were compared between patients treated with EPL versus placebo. The FAST score was calculated using data from the primary endpoint, CAP score and exploratory endpoints, LSM and AST. Additionally, the FIB‐4 index was calculated using age, AST, ALT, and platelet count to provide a non‐invasive assessment of liver fibrosis.

### 2.3 Subgroup Analysis

The change in CAP score from baseline to 6 months was assessed in subgroups categorised by baseline CAP score (< 288 and ≥ 288 dB/m) [42], HbA1c levels (< 8 and ≥ 8%), triglyceride levels (≤ 1.6935 and > 1.6935 mmol/L), country, gender, and body mass index (BMI) (< 30 and ≥ 30 kg/m^2^). Similarly, change in QoL score from baseline to 6 months was assessed in the same subgroups. Additionally, the change in CAP score from baseline to 6 months was evaluated by another baseline CAP score cut‐off (< 300 and ≥ 300 dB/m), baseline obesity classes (Class I, II and III: BMI: 30 to < 35 kg/m^2^, 35 to < 40 kg/m^2^, and ≥ 40 kg/m^2^ respectively), baseline medical history (with and without hyperlipidemia, with and without T2DM, and with and without obesity), and baseline concomitant medications (statins, glucagon‐like peptide‐1 receptor agonists [GLPI RA], and sodium‐glucose co‐transporter 2 [SGLT2] inhibitor).

### 2.4 Statistical Analysis

For power analyses, a targeted difference of 20 dB/m in CAP scores between the EPL and placebo arms after 6 months of treatment initiation was used in the sample size calculation, assuming a standard deviation of 45 dB/m. A sample size of 162 patients (*n* = 81/arm) was required for a 5% two‐sided *t*‐test to reach 80% power. Assuming a drop‐out rate of 15%, 190 patients had to be randomised (intention‐to‐treat [ITT] population) to reach the targeted number of 162 patients in the modified ITT (mITT) population. The mITT group comprised all patients from the randomization set with evaluable CAP scores at baseline and at least one postbaseline CAP measurement and who received the randomised treatment (at least 80% of the study drug planned to be given within 6 months). The changes in CAP scores from baseline to 6 months were analysed using a mixed effects model with repeated measures (MMRM) to test the null hypothesis that EPL + SoC was as efficacious in reducing steatosis as placebo + SoC. The level of statistical significance was defined as *p* < 0.05 and the null hypothesis would be rejected if *p* < 0.05. The MMRM included treatment arm, visit, country, and the treatment‐by‐visit interaction term as fixed categorical effects and the CAP score at baseline and the baseline CAP score‐by‐visit interaction were considered as covariates.

- Additionally, a linear mixed model using multiple imputations with the same fixed categorical effects and covariates as the primary MMRM was used for sensitivity analysis based on multiple imputed datasets (e.g., 100 datasets).
- All the secondary, exploratory and post hoc endpoints were analysed using MMRM, similar to the primary endpoint.

The analysis sets are described in the Table S2.

## 3 Results

### 3.1 Patient Disposition and Baseline Characteristics

**FIGURE 1:**
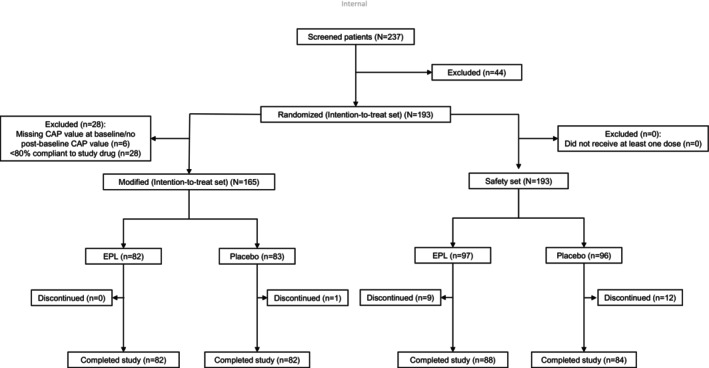
Patient disposition.

Of the 237 screened patients, 193 eligible patients were randomised (1:1; ITT set) to the EPL (*n* = 97) and placebo (*n* = 96) arm. The mITT set included 165 (85.5%) patients (*n* = 82, EPL; *n* = 83, placebo); 82 patients in each arm completed the study (Figure 1). Most of the patients in both groups were obese and had high CAP scores and mild fibrosis. Demographics and baseline characteristics in the mITT set were largely balanced between the treatment arms (Table 1). Based on medical history at baseline, both treatment arms were similar; the most common associated comorbidity was obesity followed by hyperlipidemia and T2DM. The most commonly used concomitant medication was statin (Table 1). Detailed demographics, baseline medical history, and concomitant medications of the ITT population are provided in Tables S3 and S4. Adherence to SoC (evaluated at 3 and 6 months) was high and generally balanced in both arms in the mITT set (Table S5).

**TABLE 1:** Baseline demographics and participant characteristics (mITT set).

	EPL arm (*n* = 82)	Placebo arm (*n* = 83)
Age (years); median (IQR)	56.5 (18.0)	55.0 (16.0)
Sex; *n* (%)		
Male	41 (50.0)	49 (59.0)
Female	41 (50.0)	34 (41.0)
Country; *n* (%)		
Germany	42 (51.2)	44 (53.0)
Poland	40 (48.8)	39 (47.0)
BMI (kg/m^2^)		
Median (IQR)	32 (4.8)	33.3 (5.7)
BMI category (kg/m^2^); *n* (%)		
Normal (18.5 to < 25)	3 (3.7)	2 (2.4)
Overweight (25 to < 30)	15 (18.3)	10 (12.0)
Obese (≥ 30)	64 (78.0)	71 (85.5)
Obese class I (30 to < 35)	45 (54.9)	44 (53.0)
Obese class II (35 to < 40)	16 (19.5)	19 (22.9)
Obese class III (≥ 40)	3 (3.7)	8 (9.6)
ALT (IU/L), *n*	82	83
Median (IQR)	47.5 (36.3)	48.9 (39.5)
AST (IU/L), *n*	81	82
Median (IQR)	34.7 (21.5)	34.0 (20.8)
Total cholesterol (mmol/L), *n*	82	82
Median (IQR)	4.9 (2.3)	5.2 (2.7)
Triglycerides (mmol/L), *n*	82	82
Median (IQR)	2.0 (1.2)	2.0 (1.4)
Haemoglobin A1C (%), *n*	81	83
Median (IQR)	5.8 (1.7)	5.7 (1.2)
CAP (dB/m), median (IQR)	313 (56.0)	318 (70.0)
CAP (dB/m), *n* (%)		
S1: CAP score (≥ 248 to < 268)	6 (7.3)	6 (7.2)
S2: CAP score (≥ 268 to < 280)	8 (9.8)	9 (10.8)
S3: CAP score (≥ 280)	68 (82.9)	68 (81.9)
LSM (kPa); median (IQR)	6.7 (2.3)	6.4 (2.1)
LSM (kPa); *n* (%)^a^		
F1: Mild fibrosis (5 to 7)	50 (61.0)	52 (62.7)
F2: Moderate fibrosis (7.1 to 8.8)	18 (22.0)	19 (22.9)
F3: Advanced fibrosis (8.9 to 11.6)	7 (8.5)	11 (13.3)
F4: Advanced fibrosis including cirrhosis (> 11.6)	7 (8.5)	1 (1.2)
Baseline medical history; *n* (%)		
T2DM	27 (32.9)	24 (28.9)
Hyperlipidemia	26 (31.7)	28 (33.7)
Obesity	36 (43.9)	38 (45.8)
*Concomitant medication*		
Number of subjects taking at least, *n* (%)		
Statins	33 (40.2%)	34 (41%)
GLP1 RA	6 (7.3%)	9 (10.8%)
SGLT 2 inhibitors	12 (14.6%)	5 (6%)
Number of subjects with combined medications, *n* (%)		
Statins and GLP1 RA	2 (2.4%)	7 (8.4%)
Statins and SGLT 2 inhibitors	7 (8.5%)	2 (2.4%)
GLP1 RA and SGLT 2 inhibitors	4 (4.9%)	2 (2.4%)
Statins and GLP1 RA and SGLT 2 inhibitors	2 (2.4%)	2 (2.4%)
MASLD Classification; *n* (%)		
HbA1c ≥ 8.0%	14 (17.1)	8 (9.6)
Hyperlipidemia^b^	49 (59.8)	51 (61.4)
Obesity (BMI ≥ 30 kg/m^2^)	64 (78.0)	71 (85.5)

Abbreviations: ALT, alanine transaminase; AST, aspartate transaminase; BMI, body mass index; CAP, Controlled Attenuation Parameter; EPL, essential phospholipid; HbA1c, haemoglobin A1c; IQR, interquartile range; LSM, liver stiffness measurement; MASLD, metabolic dysfunction‐associated steatotic liver disease; mITT, modified intent to treat.

^a^ Inclusion criteria allowed recruitment of patients F1 to F3 fibrosis stage as defined by LSM 5 to 13 kPa. Statistical analysis was done to include F4 based on another specific threshold [39].

^b^ Hyperlipidemia defined as baseline triglycerides > 1.6935 mmol/L.

### 3.2 Primary Endpoint

#### 3.2.1 Change in Steatosis

**FIGURE 2:**
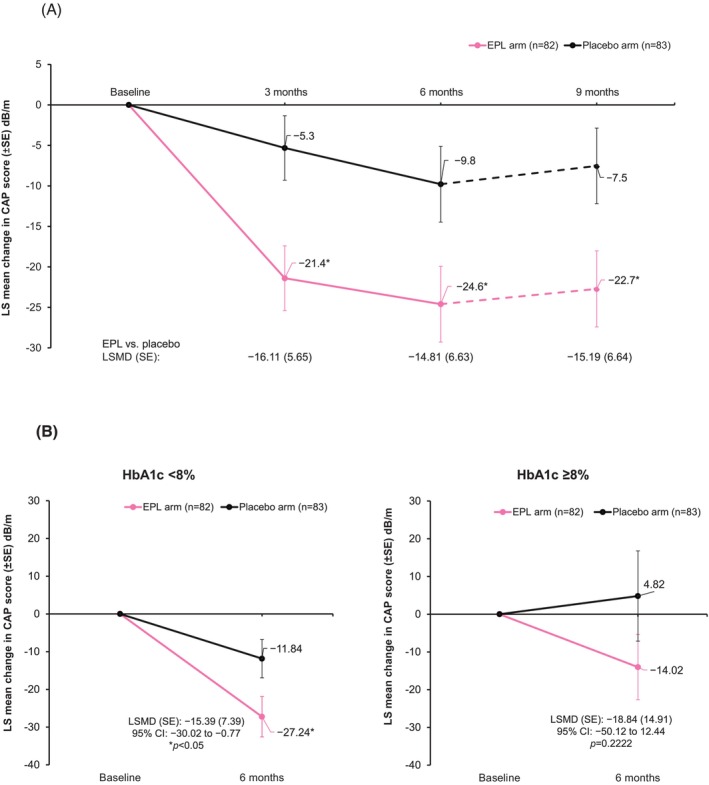
Changes in CAP score in mITT analysis set from baseline to follow‐up (A); and in subgroup by baseline HbA1c (HbA1c < 8% and HbA1c ≥ 8%) from baseline to follow‐up (B). *p < 0.05 versus placebo. The dotted line indicates 3 months follow‐up after the end of treatment.

A statistically significant reduction in the CAP score was observed in patients receiving EPL compared with those receiving placebo. At 6 months, the mean (SD) change from baseline in CAP score was −23.0 dB/m (46.50) in the EPL arm and −11.5 dB/m (42.83) in the placebo arm, with least‐square (LS) mean changes of −24.6 dB/m for the EPL arm versus −9.8 dB/m for the placebo arm, (least‐square mean difference, LSMD [standard error, SE]: −14.81 dB/m [6.63]; 95% CI: −27.89 to −1.72; *p* = 0.0269; Figure 2A). Results from the sensitivity analysis (LSMD [SE]: −14.66 dB/m [6.62]; 95% CI: −27.63 to −1.7 dB/m; *p* = 0.0267) were consistent with those from the main analysis. As a pre‐specified exploratory endpoint, the changes in CAP scores were also evaluated at 3 and 9 months. Notably, a significant improvement in steatosis with EPL treatment was observed as early as 3 months (LSMD [SE]: −16.11 dB/m [5.65]; 95% CI: −27.27 to −4.96 dB/m; *p* = 0.0049) and sustained until 3 months after the end of treatment period (LSMD [SE]: −15.19 dB/m [6.65]; 95% CI: −28.29 to −2.09 dB/m; *p* = 0.0234).

#### 3.2.2 Subgroup Analysis

When changes in CAP score at 6 months were analysed by subgroups, a statistically significant reduction in the CAP score was observed in the EPL arm versus placebo in patients with HbA1c levels < 8.0% at baseline (LSMD [SE]: −15.39 dB/m [7.39]; 95% CI: −30.02 to −0.77 dB/m; *p =* 0.0392; Figure 2B) and in patients with BMI < 30 kg/m^2^ at baseline (LSMD [SE]: −41 dB/m [18.89]; 95% CI: −79.8 to −2.19 dB/m; *p =* 0.0392; Figure 2F). In remaining subgroups, CAP score reduction at 6 months showed a numerical trend favouring EPL versus placebo (Figure S2E). In an additional analysis by another CAP score cut‐off, EPL achieved a significantly greater reduction in CAP score compared with placebo (LSMD: −18.40; *p* = 0.0253; Table S6) in the subgroup with CAP score ≥ 300 dB/m. When analysed by baseline medical history (T2DM, hyperlipidemia and obesity) EPL showed significant improvement in CAP score compared with placebo in patients without these baseline metabolic conditions (Table S7). In all remaining subgroups defined by obesity classes (Table S8) and concomitant medications (Table S9), a numerical improvement in CAP score favouring EPL versus placebo was observed.

### 3.3 Secondary Endpoints

#### 3.3.1 Change in the QoL Total Score

**FIGURE 3:**
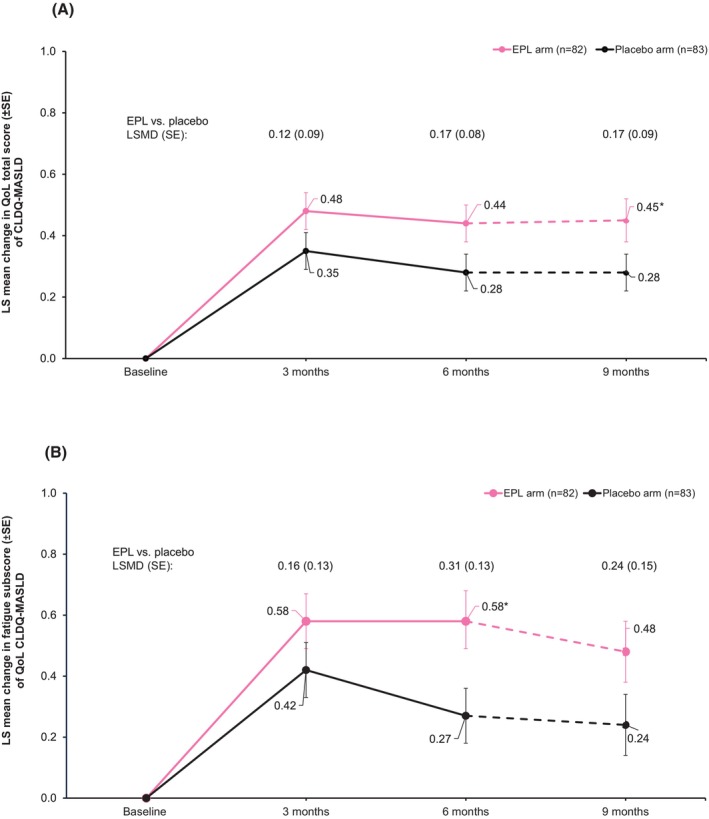
Changes in QoL total score (A), and QoL fatigue subscore (B) from baseline to follow‐up. *p < 0.05 versus placebo. The dotted line indicates 3 months follow‐up after the end of treatment.

The mean change from baseline to 6 months in the QoL total score was higher in the EPL arm than the placebo arm, although this difference was not statistically significant (LSMD [SE]: 0.17 [0.08]; *p* = 0.2445) (Figure 3A). A numerical improvement in QoL total score (exploratory analysis) was observed in the EPL arm versus placebo at 3 months (LSMD [SE]: 0.12 [0.09]; 95% CI: −0.05 to 0.3; *p* = 0.1673) and sustained at 9 months (0.17 [0.09]; 95% CI: −0.01 to 0.35; *p* = 0.0625) (Figure 3A).

When analysed by symptom subscores, treatment with EPL versus placebo demonstrated a statistically significant improvement at 6 months in fatigue subscore (LSMD [SE]: 0.31 [0.13]; 95% CI: 0.04 to 0.58; *p* = 0.0229; Figure 3B). No significant improvement was observed in other subscores with EPL treatment (Figure S3). In a subgroup of patients with baseline HbA1c levels < 8.0%, the QoL total score was significantly higher with EPL versus placebo (LSMD [SE]: 0.19 [0.09]; 95% CI: 0.01 to 0.37; *p* = 0.0381). A trend of numerical improvement was observed with EPL compared with placebo in all other subgroups.

#### 3.3.2 Changes in Symptom Evaluation

EPL treatment showed a trend toward greater improvement, though statistically non‐significant, in fatigue and asthenia versus placebo at 6 months, whereas changes in depression and abdominal pain were similar between the arms (Table S10). Changes in additional symptoms, sleep disorder, appetite loss, and irritability (analysed as exploratory endpoints) from baseline to 3, 6 and 9 months are provided in the Table S11.

### 3.4 Exploratory Efficacy Endpoints

#### 3.4.1 Change in Liver Fibrosis

At month 6, there was no statistically significant difference in liver fibrosis (measured by LSM [kPa] obtained through Fibroscan VCTE) between EPL and placebo treatment arms (LSMD [SE]: −0.06 [0.38]; 95% CI: −0.81 to 0.7; *p* = 0.8849).

### 3.5 Changes in Liver Stiffness Measurement (LSM) Category

The EPL treatment showed more improvements in LSM than placebo. A higher proportion of patients shifted from severe fibrosis to mild fibrosis in the EPL arm versus the placebo arm. By month 3 with EPL treatment, a higher proportion of patients in the EPL arm experienced a category shift in LSM from F4 and F3 to F1, compared to the placebo arm (EPL arm: 1.2% shift from F4 to F1, 3.7% shift from F3 to F1; placebo arm: 0% shift from F4 to F1, 1.2% shift from F3 to F1). At month 6, the proportion of subjects shifting from F3 to F1 was similar in both groups (4.9% in both arms), but the shift from F4 to F1 was more pronounced in the EPL arm than in the placebo (EPL arm: 2.5%, placebo arm: 1.2%). By month 9, the EPL arm again showed a higher proportion of patients shifting from F4 and F3 to F1 compared to the placebo (EPL arm: 3.7% shift from F4 to F1, 3.7% shift from F3 to F1; placebo arm: 1.2% shift from F4 to F1, 1.2% shift from F3 to F1). The LSM category changes between the baseline and month 6 are shown in Figure S4.

#### 3.5.1 Changes in CAP Score Category

Similar to the shift in LSM category, a higher proportion of patients shifted from S3 to S1 or S0 in the EPL arm versus placebo. At month 3, a higher proportion of patients in the EPL treatment arm experienced a CAP score category shift from S3 to S0 and S1, versus placebo arm (S3 to S0: 4.9%; S3 to S1: 11.0% vs. S3 to S0: 2.4%; S3 to S1: 4.8%). This trend continued, with the EPL group showing a greater shift from S3 to S0 and S1, vs. placebo at month 6 (S3 to S0: 7.4%; S3 to S1: 8.6% vs. S3 to S0: 4.9%; S3 to S1: 6.2%) and at month 9 (S3 to S0: 9.9%; S3 to S1: 8.6% vs. S3 to S0: 3.7%; S3 to S1: 7.3%). The CAP category changes between the baseline and month 6 are shown in Figure S5.

#### 3.5.2 Changes in Liver and Metabolic Parameters, and Body Weight

The mean changes in aspartate aminotransferase (AST) from baseline to 6 months was slightly higher with EPL treatment than placebo and the mean change in alanine aminotransferase (ALT), gamma‐glutamyl transferase (GGT), low‐density lipoprotein (LDL), high‐density lipoprotein (HDL), triglycerides, and total cholesterol from baseline to 6 months was mostly comparable between the treatment arms. Changes in metabolic parameters from baseline to 3, 6 and 9 months are provided in the Table S12.

**FIGURE 4:**
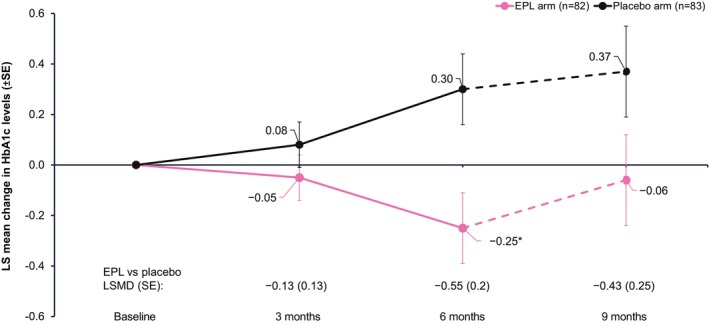
Changes in HbA1c levels from baseline to follow‐up. *p < 0.05 versus placebo. The dotted line indicates 3 months follow‐up after the end of treatment.

EPL significantly reduced the HbA1c level versus placebo at 6 months (LSMD [SE]: −0.55 [0.2]; 95% CI: −0.95 to −0.15%; *p* = 0.0069). The improvement was sustained at 9 months, though the difference did not reach statistical significance (Figure 4). No notable changes in body weight were observed at 3, 6 and 9 months in both treatment arms (Table S13).

#### 3.5.3 Patient Satisfaction With Treatment

At 6 months, more subjects in the EPL arm versus the placebo arm reported high satisfaction with therapy effectiveness (53.7% versus 50.6%), and fewer reported dissatisfaction (1.2% vs. 9.6%). However, the difference in mean change in the total satisfaction score was not statistically significant (odds ratio: 1.14; 95% CI: 0.62 to 2.1; *p* = 0.6709).

#### 3.5.4 Safety

Overall, 56.7% of patients in the ELP and 52.1% in the placebo arm reported ≥ 1 TEAE. The most common TEAEs were headache, diarrhoea, and nasopharyngitis. Overall, the safety profile of EPL was similar to that of the placebo group (Table S14).

### 3.6 Post Hoc Analysis

#### 3.6.1 FAST Score

**FIGURE 5:**
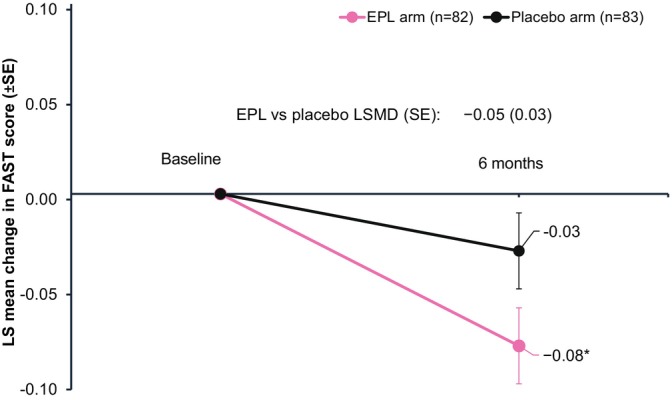
Changes in FAST score from baseline to follow‐up. *p < 0.05 versus placebo.

A significant improvement in FAST score at 6 months from baseline was observed among patients treated with EPL versus placebo. The least‐square (LS) mean changes from baseline were −0.08 for the EPL arm versus −0.03 for the placebo arm (LSMD [SE]: −0.05 [0.03]; 95% CI: −0.10 to −0.00; *p* = 0.0437; Figure 5).

#### 3.6.2 FIB‐4 Score

Additionally, for change in FIB‐4 index, a numerical trend in improvement at 6 months from baseline was observed with EPL treatment versus placebo with LSMD [SE]: −0.1 [0.08] (95% CI: −0.25 to 0.06; *p* = 0.2108; Table S15).

## 4 Conclusion

This is the first double‐blind, randomised and placebo‐controlled clinical trial that assessed the impact of EPL along with SoC on liver steatosis and QoL in patients with MASLD who had the metabolic comorbidities T2DM, hyperlipidemia, or obesity. The study met its primary endpoint by showing a statistically significant reduction in the CAP score from baseline to month 6 with EPL treatment compared to placebo. This finding is in agreement with results from previous studies using abdominal ultrasound [31]. The beneficial effects of EPL on CAP score were observed as early as 3 months and sustained for up to 9 months, including 3 months post‐treatment. Notably, across subgroups defined by CAP cut‐offs (< and ≥ 288 dB/m; < and ≥ 300 dB/m), EPL showed statistically significant benefit in those with more severe baseline steatosis (≥ 300 dB/m). Additionally, more patients receiving EPL improved from steatosis category S3 to S1 and S0 than those receiving placebo, reaffirming a potential clinical benefit of EPL in improving CAP scores over time. Importantly, these results were observed in a study population wherein most patients were in the highest steatosis category (S3) at baseline with CAP scores of 280 dB/m or higher. Subgroup analysis showed significantly greater improvement in steatosis with EPL treatment than with placebo in a subgroup of patients with baseline HbA1c levels < 8% and BMI < 30 kg/m^2^. EPL showed a numerical but non‐significant improvement in CAP score compared with placebo in participants with obesity and across obesity classes I and II.

Supporting these results, EPL significantly improved HbA1c levels compared with placebo over 6 and 9 months, indicating a notable therapeutic benefit of EPL in glycemic control. Interestingly, changes in body weight between the EPL and the placebo groups did not differ, indicating a direct effect of EPLs on reducing hepatic steatosis.

Such an impact of EPL on the liver is supported by knowledge of the underlying mechanism of action of EPL at the cellular level. EPL was found to significantly increase hepatocyte membrane fluidity and increase hepatocellular export of lipids in steatotic HepaRG cells [36]. Depletion of phospholipids from the cell membrane can impact protein transport and lipid accumulation in the hepatocytes [36]. PC, a key ingredient of EPL, was found to prevent fatty infiltration by reducing triglyceride production through the suppression of fatty acid synthesis and the enhancement of β‐oxidation in animal models [43, 44]. A recent study found that fatty acid synthase in steatotic HepaRG cells had significantly decreased with EPL treatment [37]. EPL is also found to play a role in facilitating lipid export via very‐low‐density lipoprotein formation and elimination via PPARα activation [45]. In another recently presented study, EPL treatment significantly reduced lipid droplet size in steatotic hepatocytes [46]. These mechanisms could collectively contribute to the reduction of liver steatosis by EPL treatment, as observed in the present study.

MASLD is increasingly recognised as symptomatic, with early‐stage patients often experiencing subtle non‐specific symptoms that potentially affect PROs and negatively impact HRQoL [16]. Abdominal discomfort and fatigue were reported by gastroenterologists and general physicians as occurring ‘very often’ in a real‐world study [5]. In this study, along with steatosis improvement, EPL treatment showed a numerical improvement in QoL total score, with statistical significance in patients with Hba1c level < 8%. Notably, in the present study treatment with EPL versus placebo resulted in a statistically significant improvement in fatigue subscore and a numerical improvement in abdominal subscore, the predominant symptoms contributing to the QoL burden. Symptom improvement is not frequently reported across studies. Ivaskin et al. reported a significant improvement in gastrointestinal symptom score after 12 weeks of EPL treatment [47]. The observed improvement in abdominal pain subscore in that study could be related to steatosis reduction observed with EPL treatment; however, the mechanism behind fatigue improvement remains unclear. Addressing fatigue is crucial and its alleviation with EPL treatment could enhance patient engagement in physical activities leading to better MASLD management.

At month 6, change in transaminase levels was comparable between the treatment arms, while HDL‐cholesterol levels slightly increased and LDL‐cholesterol levels decreased with EPL versus placebo, although the differences were not statistically significant. EPL treatment was associated with a minor and non‐significant fibrosis improvement (by LSM), with more patients shifting from category F4/F3 to F1 versus placebo. Additionally, EPL showed a modest trend toward improvement in FIB‐4 at 6 months compared with placebo, though not statistically significant. The short treatment duration may explain the lack of statistical significance regarding the observed difference. The observed reduction in hepatic steatosis and HbA1c levels with EPL suggests potential benefits in fibrosis reduction with longer treatment. Effective management of MASLD necessitates not only good glycemic control but also adequate weight management. In the present study, EPL treatment did not affect weight, unlike PPARγ or pan‐PPAR agonists [48]. Of note, EPL‐treated patients had a significant improvement in FAST score compared to placebo at 6 months, suggesting a potential benefit in lowering the risk of fibrosis progression. While the magnitude of improvement appears modest, even small reductions in fibrosis risk could translate to meaningful clinical benefit, given the progressive nature of MASLD.

The treatment landscape for MASLD is multifaceted and rapidly evolving. Lifestyle interventions remain the cornerstone of MASLD management [9, 19, 20, 21], while pharmacological treatments are increasingly explored to address the complex pathophysiology of MASLD [12, 49, 50, 51]. Resmetirom, a THR‐β agonist, was recently approved for MASH with moderate to advanced fibrosis [22, 23], though no approved therapies exist for early steatosis management yet. Emerging therapies targeting hepatic lipid accumulation, oxidative stress, and inflammation are also being studied for their potential to mitigate liver damage. EPL offers a promising adjunctive treatment option that can be integrated with existing pharmacological and lifestyle interventions. Moreover, EPL stands out due to its low cost, supporting broader access and long‐term adherence.

The study is limited by the relatively short duration of the intervention and the choice of the non‐invasive endpoint. Moreover, it remains to be determined if a CAP score reduction is associated with an improvement of long‐term clinical endpoints. A strength of the study is the inclusion of mostly people with MASLD, who are obese and have T2DM, but not yet have severe liver disease. This population is representative for most people with MASLD and for which the Consensus Report of the American Diabetes Association most recently provided recommendations for screening, risk stratification, and treatment [6].

In conclusion, given the observed beneficial effects of EPL in this phase 4 clinical trial, including anti‐steatotic effects, improved QoL measures, improved glycemic control, and a good safety profile in patients with non‐cirrhotic MASLD and T2DM, hyperlipidemia, or obesity, EPL could add to the growing armamentarium in the comprehensive management of steatosis, contributing to the overall liver health and patients' well‐being.

The Supporting Information accompanying this article includes additional data and figures that support the findings of this study.

## Author Contributions

N.S., M.H., J.F. and M.D. were involved in conceptualization, investigation, methodology, resources management, supervision, validation, visualisation, original draft preparation and subsequent review; J.M.S. was involved in conceptualization, investigation, methodology, validation, visualisation, original draft preparation and subsequent review; J.G. and L.B. were involved in investigation, methodology, resources management, supervision, validation, visualisation, original draft preparation and subsequent review; B.P. was involved in conceptualization, formal analysis, funding acquisition, methodology, project administration, validation, visualisation, original draft preparation, and subsequent review; B.B.D.F. was involved in data curation, formal analysis, methodology, project administration, software, validation, visualisation, original draft preparation, and subsequent review; R.V. was involved in methodology, project administration, validation, visualisation, original draft preparation, and subsequent review; all authors approved the final version of the manuscript.

## Funding

This work was supported by Opella.

## Ethics Statement

The trial was approved by the Ethics Committee at the Medical Faculty of the Eberhard Karls University and University Hospital Tübingen, Germany.

## Consent

All patients in this trial provided written informed consent.

## Conflicts of Interest

N.S. has received research support and materials from Opella to conduct the study; fees for consultancy and scientific talks from Allergan, AstraZeneca, Boehringer Ingelheim, Gilead, Genkyotex, GSK, Intercept Pharma, Lilly, Merck Sharpe & Dohme, Novartis, Novo Nordisk, and Pfizer; and received research support from AstraZeneca, Boehringer Ingelheim, DSM Nutritional Products, and Roche Diagnostics; and serves as coleader in the German Diabetes Association Study group MASLD. M.D. has received speaker honorary from Gilead Sciences, AbbVie, Falk, Advanz, Ipsen, Merz, Boehringer, Astra Zeneca, and Opella; honoraria for advisory boards from Gilead Sciences, Advanz, Ipsen, Boehringer, GSK, and Opella; travel grants from Gilead, AbbVie, Advanz, and Ipsen; and research funding from Merz, and Echosens. J.M.S. has received consultant honorary from Akero, Alentis, Alexion, Altimmune, Astra Zeneca, 89Bio, Bionorica, Boehringer Ingelheim, Boston Pharmaceuticals, Gilead Sciences, GSK, HistoIndex, Ipsen, Inventiva Pharma, Madrigal Pharmaceuticals, PRO.MED.CS Praha a.s., Kríya Therapeutics, Eli Lilly, MSD Sharp & Dohme GmbH, Novartis, Novo Nordisk, Pfizer, Roche, Sanofi, and Siemens Healthineers; speaker honoraria from AbbVie, Boehringer Ingelheim, Gilead Sciences, Ipsen, Eli Lilly, Novo Nordisk, and Madrigal Pharmaceuticals; and has stockholder options in Hepta Bio. J.G. has received lecture honoraria from Opella. B.P., R.V. and B.B. are employees of Opella and may hold shares and/or stock options in the company. M.H., J.F. and L.B. declare no conflicts of interest.

## Supporting information


**Data S1:** liv70601‐sup‐0001‐supinfo.docx.

**Data S2:** liv70601‐sup‐0002‐supinfo.docx.

## Data Availability

The data that support the findings of this study are available on request from the corresponding author. The data are not publicly available due to privacy or ethical restrictions.
