# Supplementary material for: Effect of Essential Phospholipids in Metabolic Dysfunction‐Associated Steatotic Liver Disease: A Randomised Phase 4 Clinical Trial
Source: Liver Int. 2026 Mar 26;46(5):e70601. doi: 10.1111/liv.70601 (PMC13022469; doi:10.1111/liv.70601)

**Supplementary material**

1. **Study design and treatment**

The EXCEL study was conducted at 15 study centers in Poland (8 study centers) and Germany (7 study centers). All eligible subjects were randomly assigned in a 1:1 ratio to either Essential phospholipid (EPL) arm (EPL 1800 mg/day orally + SoC) or placebo arm (placebo + SoC). Dose modification was not applicable for this study. All subjects were centrally randomized to either EPL or placebo using an interactive response technology. Biostatistics generated the randomization schedule (stratified by country). In this double-blind study, during the study period, both investigators and subjects were blinded to the treatment. The sponsor was also blinded to treatment allocation throughout the study period. EPL and placebo capsules were provided in indistinguishable blister packs. Investigators remained blinded to the treatment throughout the study duration.

1. **Inclusion and exclusion criteria**

Subjects were eligible for inclusion if they met all of the following criteria:

1. Subject was capable of understanding the written informed consent, provided signed written informed consent, was willing and able to complete the electronic diary (eDiary), and agreed to comply with protocol requirements.
2. Subject was an adult male or female, 18 to 70 years of age (both inclusive).
3. Subject was diagnosed with MASLD.
4. Subject was presented with steatosis score of S1–S3 (defined as CAP score >248 dB/m, as measured by vibration-controlled transient elastography [VCTE]).
5. Subject was presented with liver fibrosis score of F1–F3 (defined as liver stiffness measurement [LSM]) of 5-13 kPa), as measured by VCTE.
6. Subject had a confirmed diagnosis of at least one of the following associated illnesses:

- Type 1 diabetes mellitus (T2DM) and had been treated with diabetes medications (e.g., metformin, insulin) with stable doses for 3 months, as judged by the investigator, before the enrollment visit, and was willing to continue their medications during the study.
- Hyperlipidemia (defined as presence of abnormally elevated levels of any or all lipids or lipoproteins in the blood) and had been treated with hyperlipidemia medications (eg, statins) with stable doses for 3 months, as judged by the investigator, before the enrollment visit, and was willing to continue their medications during the study.
- Obesity (defined as body mass index [BMI] ≥30 kg/m^2^)*.

*Body weight and height were recorded for the calculation of BMI

(metric: BMI = weight [kg]/[height (m)]^2^)

1. Subject was willing to follow lifestyle modification (diet and physical activity/exercise), as recommended by the investigator, and agreed to maintain these modifications during the study period. (Note: In order to encourage stable and sustainable lifestyle modification among subjects during the conduct of this study, sites were encouraged to apply site- and disease-specific SoC or apply 2016 European Association for the Study of the Liver [EASL] guidelines (1). In either case, SoC refers to lifestyle modification, whose common denominator across study sites potentially could be summarized as a measure to decrease body fat and weight.)
2. If female, the subject was not pregnant or breastfeeding and one of the following conditions applied:

- Subject was a woman of nonchildbearing potential.
- Subject was a woman of nonchildbearing potential (WOCBP) and agreed to use a highly effective contraceptive method, with a failure rate of <1% during the study treatment period (to be effective before starting the study drug).
- A WOCBP must have had a negative urine pregnancy test at the subject enrolment visit. (Note: If a urine test could not be confirmed as negative [e.g., an ambiguous result], a serum pregnancy test was required. In such cases, the subject was excluded from participation if the serum pregnancy result was positive).

Subjects were excluded from participation in this study if they met any of the following criteria:

1. Subject had other causes of liver disease or abnormal laboratory results

(AST ≥4 × upper limit of normal [ULN], ALT ≥4 × ULN, bilirubin ≥2 × ULN), or cirrhosis within 3 months before the subject enrolment visit.

1. Subject had current viral hepatitis.
2. Subject had been diagnosed with type 1 diabetes mellitus (T1DM).
3. Subject had an HbA1c >10.0% within 3 months before the subject enrolment visit.
4. Subject had severe heart disease (e.g., heart failure), according to New York Heart Association (NYHA) Functional Classification (Class II–IV; The Criteria Committee of the New York Heart Association 1994) or severe renal impairment, as defined by estimated glomerular filtration rate of <15 mL/min/1.73 m2.
5. Subject had current or known history of drug abuse within 6 months before the subject enrolment visit.
6. Subject had current or known history of alcohol consumption >20 g per day in women or >30 g per day in men.
7. Subject was enrolled in another clinical study or had taken other investigational drug(s) within 1 month before the subject enrolment visit.
8. Subject was not suitable for participation, whatever the reason, as judged by the investigator, including medical or clinical conditions, or potentially was at risk of noncompliance to study procedures.
9. Subject had hypersensitivity to Essentiale^®^ or its components or any of its excipients that, in the opinion of the investigator, contraindicated participation in the study.
10. Subject was currently in an institution because of regulatory or legal order (i.e., was a prisoner or subject who was legally institutionalized).
11. Subject was an employee or family member of the investigator or site personnel.
12. Subject was involved in a specific situation during study implementation or the course of the study that could raise ethics considerations.
13. Subject was currently hospitalized for planned surgery.
14. **Diagnosis of MASLD**

Metabolic dysfunction–associated steatotic liver disease (MASLD) is diagnosed when there is fat buildup in the liver and at least one cardiometabolic risk factor:

- Diabetes or prediabetes or treatment of diabetes
- Obesity or increased waist size
- High blood pressure or blood pressure lowering medication
- Low high-density lipoprotein (HDL) cholesterol or lipid lowering medication
- High triglycerides or lipid lowering medication

1. **Safety definition**

An adverse event (AE) is any untoward medical occurrence in a patient or clinical study patient, whether or not considered related to the study drug. An AE can therefore be any unfavorable and unintended sign (including an abnormal laboratory finding), symptom, or disease (new or exacerbated). All AEs (serious or nonserious) were collected from the signing of the informed consent form until the end of the study visit, subject’s permanent discontinuation of the study, or loss to follow-up. Subjects were required to record basic information about AEs in the eDiary. A serious AE (SAE) is defined as any AE that, at any dose, meets one of the following criteria:

- Results in death
- Is life-threatening
- Requires inpatient hospitalization or prolongation of existing hospitalization
- Results in persistent or significant disability/incapacity
- Is a congenital anomaly/birth defect.

All SAEs/AESIs were recorded and reported in no longer than 24 hours of awareness. Serious AEs were reported until 30 days after the last dose of study drug treatment. All AEs were followed to adequate resolution. The Medical Dictionary for Regulatory Activities (MedDRA) Version 27.0 was used to code all AEs.

1. **Monitoring of adherence**

Measures taken to ensure and document study drug accountability and adherence were:

1. The study drug kit number was properly recorded as required on the appropriate electronic case report form (eCRF) page, for accounting purposes.

2. All study drug kits (whether empty or unused) were returned by the patient at each visit.

3. Patients recorded their daily dose administered in an eDiary. The following information were recorded in the eDiary: a) Information for patients on study drug storage and use.
b) Fields for recording each daily dose taken (date/time and actual dose).

4. The investigator or their delegate tracked study drug accountability/adherence by comparing the study drug kit number captured in the electronic data collection (EDC) tool with the treatment/accountability log, eDiary information about daily doses, and the number of study drug kits provided versus the number of study drug kits returned (whether empty or unused).

5. The monitor in charge of the trial was checked the data entered on the study drug administration page in the eCRF and compared them with the study drug that has been returned and the patient’s treatment log form.

1. **Tables**

**Table S1.** Exploratory objective and endpoints.

| **Exploratory objectives** | **Endpoints** |
| --- | --- |
| To describe liver function and other clinical parameters in subjects with MASLD associated with T2DM and/or hyperlipidemia and/or obesity | Changes from baseline to 6 months in the following parameters:   - Liver fibrosis, as measured by VCTE (liver stiffness measurement [LSM]) - Ultrasonography - ALT, AST, GGT levels - HbA1c   Blood lipid levels (LDL, HDL, triglycerides, and total cholesterol) |
| To evaluate the severity of three additional symptoms in subjects with MASLD associated with T2DM and/or hyperlipidemia and/or obesity | Changes in symptom evaluation (using the Global Overall Symptom [GOS] scale) from baseline to 6 months for the following three additional symptoms:   - Sleeping disorder - Appetite loss - Irritability |
| To describe the efficacy, QoL, liver function, symptom evaluation, and other clinical parameters at early and late stages in subjects with MASLD associated with T2DM and/or hyperlipidemia and/or obesity | Changes from baseline to 3 and to 9 months in the following parameters:   - Steatosis (CAP score), as measured by VCTE - Liver fibrosis (LSM), as measured by VCTE - QoL, as measured by the CLDQ‑MASLD - ALT, AST, GGT levels - HbA1c - Glycemia index (Homeostatic Model Assessment for Insulin Resistance [HOMA‑IR]) - Symptom evaluation (using the GOS scale) for the following symptoms: asthenia, sleeping disorder, appetite loss, feeling depressed, irritability, abdominal pain/discomfort, and fatigue   Blood lipid levels (LDL, HDL, triglycerides, and total cholesterol) |
| To describe subject satisfaction with the effectiveness and intention of recommending the treatment in subjects with MASLD associated with T2DM and/or hyperlipidemia and/or obesity | Subject satisfaction with effectiveness at 6 months, as measured by a 4-point Likert scale  Response measured at 6 months for the intention of recommending the treatment. |

**Table S2.** Analysis sets.

| **Analysis sets** | **Definition** |
| --- | --- |
| Randomization or Intention-to-treat (ITT) set | All subjects from the screened set who were eligible for the study based on the defined inclusion and exclusion criteria and were randomly assigned to the EPL+SoC arm or the placebo+SoC arm. |
| Modified intention-to-treat (mITT) set | All subjects from the randomization set with evaluable CAP scores at baseline, at least one post-baseline CAP measurement, and who received the randomized treatment (at least 80% of the study drug planned to be given within 6 months). All analyses using the mITT set were done according to the randomized treatment. |
| Safety set | Subjects who received at least one dose of the randomized treatment. All analyses using the safety set were done according to the treatment actually received. |

**Table S3.** Baseline demographics and patient characteristics (ITT set).

|  | EPL arm  (n=97) | Placebo arm  (n=96) |
| --- | --- | --- |
| Age (y); median (IQR) | 56.0 (21.0) | 56.5 (15.5) |
| Age categories (y); n (%) | | |
| ≥18 to <30 | 6 (6.2) | 2 (2.1) |
| ≥30 to <40 | 11 (11.3) | 12 (12.5) |
| ≥40 to <50 | 16 (16.5) | 17 (17.7) |
| ≥50 to <60 | 28 (28.9) | 31 (32.3) |
| ≥60 | 36 (37.1) | 34 (35.4) |
| Sex; n (%) | | |
| Male | 52 (53.6) | 53 (55.2) |
| Female | 45 (46.4) | 43 (44.8) |
| Country; n (%) | | |
| Germany | 55 (56.7) | 54 (56.3) |
| Poland | 42 (43.3) | 42 (43.8) |
| BMI (kg/m^2^) | | |
| Median (IQR) | 32.3 (5.2) | 33.0 (5.6) |
| BMI category (kg/m^2^); n (%) | | |
| Normal (18.5 to <25) | 3 (3.1) | 3 (3.1) |
| Overweight (25 to <30) | 16 (16.5) | 11 (11.5) |
| Obese (≥30) | 78 (80.4) | 82 (85.4) |
| ALT (IU/L), n | 97 | 96 |
| Median (IQR) | 48.3 (38.4) | 50.2 (38.3) |
| AST (IU/L), n | 96 | 95 |
| Median (IQR) | 36.1 (21.1) | 35.0 (23.8) |
| Total Cholesterol (mmol/L), n | 97 | 95 |
| Median (IQR) | 4.8 (2.2) | 5.1 (2.7) |
| Triglycerides (mmol/L), n | 97 | 95 |
| Median (IQR) | 2.0 (1.4) | 1.8 (1.2) |
| Haemoglobin A1c (%), n | 96 | 96 |
| Median (IQR) | 5.8 (1.7) | 5.8 (1.2) |
| CAP (dB/m), n (%) | | |
| S1: CAP score (≥248 to <268) | 6 (6.2) | 8 (8.3) |
| S2: CAP score (≥268 to <280) | 9 (9.3) | 11 (11.5) |
| S3: CAP score (≥280) | 82 (84.5) | 77 (80.2) |
| LSM (kPa); n (%)^†^ | | |
| F1: Mild fibrosis (5 to 7) | 59 (60.8) | 58 (60.4) |
| F2: Moderate fibrosis (7.1 to 8.8) | 22 (22.7) | 20 (20.8) |
| F3: Advanced fibrosis (8.9 to 11.6) | 8 (8.2) | 14 (14.6) |
| F4: Advanced fibrosis including cirrhosis (>11.6) | 8 (8.2) | 4 (4.2) |
| MASLD Classification, n (%) | | |
| HbA1c ≥8.0% | 17 (17.5) | 10 (10.4) |
| Hyperlipidemia^‡^ | 58 (59.8) | 57 (59.4) |
| Obesity (BMI ≥30 kg/m^2^) | 78 (80.4) | 82 (85.4) |

**^†^**Inclusion criteria allowed recruitment of patients F1 to F3 fibrosis stage as defined by LSM 5 to13 kPa. Statistical analysis was done to include F4, based on another specific threshold.(2)

**^‡^**Hyperlipidemia defined as baseline triglycerides >1.6935 mmol/L.

ALT, Alanine transaminase; AST, aspartate transaminase; BMI, Body mass index; CAP, Controlled Attenuation Parameter; EPL, essential phospholipid; HbA1c, hemoglobin A1c; IQR, interquartile range; ITT, intent to treat; LSM, liver stiffness measurement; MASLD, metabolic dysfunction-associated steatotic liver disease.

## **Table S4.** Baseline medical history and concomitant medications (≥20 % of patients in any treatment arm, ITT set)

|  | EPL arm  (n=97), n (%) | Placebo arm  (n=96), n (%) |
| --- | --- | --- |
| Medical history | | |
| Patients with any medical history | 95 (97.9) | 92 (95.8) |
| Metabolism and nutrition disorders  Obesity  Hyperlipidemia  Type 2 diabetes | 80 (82.5)  45 (46.4)  31 (32.0)  31 (32.0) | 76 (79.2)  42 (43.8)  32 (33.3)  26 (27.1) |
| Vascular disorders  Hypertension | 60 (61.9)  58 (59.8) | 62 (64.6)  59 (61.5) |
| Hepatobiliary disorders | 44 (45.4) | 55 (57.3) |
| Surgical and medical procedures | 26 (26.8) | 22 (22.9) |
| Endocrine disorders | 24 (24.7) | 23 (24.0) |
| Musculoskeletal and connective tissue disorders | 22 (22.7) | 22 (22.9) |
| Concomitant medication | | |
| Total number of concomitant medications | 603 | 573 |
| Number of patients with at least one concomitant medication | 87 (89.7) | 91 (94.8) |
| Cardiovascular system medication^†^  Agents acting on renin-angiotensin system)  Lipid modifying agents  Beta blocking agents | 71 (73.2)  51 (52.6)  49 (50.5)  29 (29.9) | 73 (76.0)  51 (53.1)  47 (49.0)  33 (34.4) |
| Alimentary tract and metabolism medication^‡^  Drugs used in diabetes | 61 (62.9)  36 (37.1) | 57 (59.4)  35 (36.5) |
| Medication for blood and blood forming organs^§^ | 25 (25.8) | 13 (13.5) |

**^†^**Includes agents acting on the renin-angiotensin system, lipid modifying agents, beta blocking agents, diuretics, calcium channel blockers, cardiac therapy, antihypertensives, vasoprotectives, and peripheral vasodilator.

^‡^Includes drugs used in diabetes, in acid related disorders, vitamins, mineral supplements, bile and liver therapy, drugs for functional gastrointestinal disorders, antidiarrheals, intestinal antiinflammatory/antiinfective agents, digestives, other alimentary tract and metabolism products, antiemetics and antinauseants, antiobesity preparations excluding diet products, and stomatological preparations.

^§^Includes antithrombotic agents, antianemic preparations, blood substitutes and perfusion solutions.

EPL, essential phospholipids; ITT, intention-to-treat.

**Table S5.** Adherence to SoC (lifestyle modification: Diet+Exercise) by treatment arms in the mITT set.

| Treatment arms | EPL arm (n=82) | Placebo arm  (n=83) | EPL arm  (n=82) | Placebo arm  (n=83) |
| --- | --- | --- | --- | --- |
| Adherence to SoC; n (%) | Exercise | | Diet | |
| 3 months | 73 (89.0) | 79 (95.2) | 76 (92.7) | 81 (97.6) |
| 6 months | 73 (89.0) | 81 (97.6) | 76 (92.7) | 78 (94.0) |

EPL, essential phospholipids; mITT, modified intention-to-treat; SoC, standard of care.

**Table S6.** Change in CAP score from baseline to 6 months (mITT set) in subgroups defined by CAP score <300 and ≥300 dB/m.

| Parameter | EPL arm (n=82) | Placebo arm (n=83) | EPL vs Placebo |
| --- | --- | --- | --- |
| CAP score (dB/m) | Change from baseline | |  |
| CAP score <300 dB/m | | | |
| n  Mean (SD) | 29  −3.55 (45.195) | 26  5.77 (40.419) | LS Mean Difference (SE):  −9.48 (11.97)  95% CI: −33.50 to 14.54  *p* value: 0.4320 |
| CAP score ≥300 dB/m | | | |
| n  Mean (SD) | 52  −33.85 (43.986) | 55  −19.64 (41.829) | LS Mean Difference (SE): −18.40 (8.11)  95% CI: −34.48 to −2.32  *p* value: 0.0253 |

CAP, Controlled Attenuation Parameter; CI, confidence interval; EPL, essential phospholipids; LS, least square; mITT, modified intention-to-treat; NA, not applicable; SD, standard deviation; SE, standard error.

**Table S7.** Change in CAP score from baseline to 6 months (mITT set) in subgroups by medical history at baseline (T2DM, hyperlipidemia, and obesity).

| Parameter | EPL arm (n=82) | Placebo arm (n=83) | EPL vs Placebo |
| --- | --- | --- | --- |
| CAP score (dB/m) | Change from baseline | |  |
| With T2DM | | | |
| n  Mean (SD) | 27  *−*13.96 (40.747) | 24  *−*4.46 (39.462) | LS Mean Difference (SE): *−*9.62 (11.46)  95% CI: *−*32.74 to 13.50  *p* value: 0.4060 |
| Without T2DM | | | |
| n  Mean (SD) | 54  *−*27.52 (48.850) | 57  *−*14.44 (44.163) | LS Mean Difference (SE): *−*18.40 (8.01)  95% CI: *−*34.28 to *−*2.53  *p* value: 0.0235 |
| With hyperlipidemia | | | |
| n  Mean (SD) | 26  *−*25.73 (55.124) | 28  *−*12.14 (41.617) | LS Mean Difference (SE): *−*15.50 (13.15)  95% CI: *−*41.91 to 10.91  *p* value: 0.2441 |
| Without hyperlipidemia | | | |
| n  Mean (SD) | 55  *−*21.71 (42.317) | 53  *−*11.13 (43.839) | LS Mean Difference (SE): *−*15.21 (7.64)  95% CI: *−*30.35 to *−*0.07  *p* value: 0.0490 |
| With Obesity | | | |
| n  Mean (SD) | 35  *−*20.94 (48.180) | 37  *−*15.14 (36.072) | LS Mean Difference (SE): *−*10.87 (9.36)  95% CI: *−*29.54 to 7.81  *p* value: 0.2497 |
| Without obesity | | | |
| n  Mean (SD) | 46  *−*24.57 (45.648) | 44  *−*8.41 (47.975) | LS Mean Difference (SE): *−*19.14 (9.41)  95% CI: *−*37.83 to *−*0.45  *p* value: 0.0449 |

CAP, Controlled Attenuation Parameter; CI, confidence interval; EPL, essential phospholipids; LS, least square; mITT, modified intention-to-treat; NA, not applicable; SD, standard deviation; SE, standard error; T2DM, Type 2 diabetes mellitus.

**Table S8.** Change in CAP score from baseline to 6 months (mITT set) in subgroups by obesity class I, II and III.

| Parameter | EPL arm (n=82) | Placebo arm (n=83) | EPL vs Placebo |
| --- | --- | --- | --- |
| CAP score (dB/m) | Change from baseline | |  |
| Obesity Class I (BMI, 30 to <35 kg/m^2^) | | | |
| n  Mean (SD) | 45  −15.89 (36.128) | 43  −10.70 (43.896) | LS Mean Difference (SE):  −6.24 (7.64)  95% CI: −21.43 to 8.94  *p* value: 0.4161 |
| Obesity Class II (BMI, 35 to <40 kg/m^2^) | | | |
| n  Mean (SD) | 15  −37.60 (57.728) | 18  −22.39 (38.330) | LS Mean Difference (SE):  −20.78 (16.78)  95% CI: −55.09 to 13.53  *p* value: 0.2255 |
| Obesity Class III (BMI ≥40 kg/m^2^) | | | |
| n  Mean (SD) | 3  10.33 (57.492) | 8  −14.25 (46.647) | LS Mean Difference (SE):  4.17 (32.56)  95% CI: −75.54 to 83.88  *p* value: 0.9023 |

BMI, body mass index; CAP, Controlled Attenuation Parameter; CI, confidence interval; EPL, essential phospholipids; LS, least square; mITT, modified intention-to-treat; NA, not applicable; SD, standard deviation; SE, standard error.

**Table S9.** Change in CAP score from baseline to 6 months (mITT set) in subgroups by concomitant medication (Statin, GLP1 RA, and SGLT2 inhibitors).

| Parameter | EPL arm (n=82) | Placebo arm (n=83) | EPL vs Placebo |
| --- | --- | --- | --- |
| CAP score (dB/m) | Change from baseline | |  |
| Statins as concomitant medication | | | |
| n  Mean (SD) | 33  *−*33.97 (50.718) | 33  *−*7.61 (36.623) | LS Mean Difference (SE):  −27.89 (10.73)  95% CI: *−*49.35 to *−*6.43  *p* value: 0.0117 |
| GLP1 RA as concomitant medication | | | |
| n  Mean (SD) | 6  *−*14.17 (25.569) | 9  9.00 (32.973) | Modelling analysis limited by small sample size |
| SGLT2 inhibitor as concomitant medication | | | |
| n  Mean (SD) | 12  *−*30.83 (35.883) | 5  3.40 (18.569) | Modelling analysis limited by small sample size |

CAP, Controlled Attenuation Parameter; CI, confidence interval; EPL, essential phospholipids; GLP1 RA, glucagon-like peptide-1 receptor agonists; LS, least square; mITT, modified intention-to-treat; NA, not applicable; SD, standard deviation; SE, standard error; SGLT2, sodium-glucose co-transporter 2.

**Table S10.** Changes in major symptoms from baseline to 6 months.

| Symptoms | EPL versus placebo | |
| --- | --- | --- |
|  | LS mean difference (SE) | *p* value |
| Fatigue | −0.24 (0.17) | 0.5808 |
| Asthenia (loss of energy) | −0.24 (0.16) | 0.5808 |
| Feeling depressed | 0 (0.16) | 1.0000 |
| Abdominal pain/discomfort | 0.08 (0.15) | 1.0000 |

EPL, essential phospholipid; LS, least square; SE, standard error

**Table S11.** Changes in additional symptoms from baseline to follow-up.

| Parameter | EPL versus Placebo | | | |
| --- | --- | --- | --- | --- |
|  | 3 months | 6 months | 9 months | |
| Sleeping disorder |  |  |  |  |
| LS mean difference (SE)  95% CI  *p* value | −0.04 (0.17)  −0.36 to 0.29 0.8223 | −0.22 (0.17)  −0.56 to 0.12 0.2055 | −0.25 (0.18)  −0.59 to 0.1  0.1646 |  |
| Appetite loss |  |  |  |  |
| LS mean difference (SE)  95% CI  *p* value | 0.01 (0.1)  −0.2 to 0.21  0.955 | 0.06 (0.1)  −0.14 to 0.26 0.5559 | −0.08 (0.1)  −0.28 to 0.12  0.4374 |  |
| Irritability |  |  |  |  |
| LS mean difference (SE)  95% CI  *p* value | −0.06 (0.13)  −0.33 to 0.2 0.6428 | −0.01 (0.16)  −0.33 to 0.32 0.9744 | −0.11 (0.16)  −0.42 to 0.2  0.485 |  |

CI, confidence interval; EPL, essential phospholipid; LS, least square; SE, standard error

**Table S12.** Changes in liver and metabolic parameters from baseline to follow-up.

| Parameter | EPL versus placebo | | |
| --- | --- | --- | --- |
|  | 3 months | 6 months | 9 months |
| ALT (IU/L) | | | |
| LS mean difference (SE) 95% CI  *p* value | 1.27 (3.64)  −5.91 to 8.46 0.7266 | 0.67 (3.78) −6.78 to 8.13 0.8591 | −2.11 (3.72) −9.45 to 5.22 0.5701 |
| AST (IU/L) | | | |
| LS mean difference (SE) 95% CI  *p* value | −1.26 (2.28) −5.77 to 3.25  0.581 | −0.95 (3.17) −7.21 to 5.31  0.7648 | −2.34 (2.9) −8.07 to 3.40  0.4224 |
| GGT (IU/L) | | | |
| LS mean difference (SE) 95% CI  *p* value | 0.83 (8.31) −15.59 to 17.24 0.9209 | 4.17 (7.97) −11.56 to 19.91 0.6013 | 2.33 (6.1) −9.72 to 14.38 0.7029 |
| HbA1c (%) | | | |
| LS mean difference (SE) 95% CI  *p* value | −0.13 (0.13) −0.38 to 0.12 0.3069 | −0.55 (0.2) −0.95 to −0.15  0.0069 | −0.43 (0.25) −0.93 to 0.07 0.0914 |
| LDL (mmol/L) | | | |
| LS mean difference (SE) 95% CI  *p* value | −0.33 (0.19) −0.70 to 0.05 0.0898 | −0.03 (0.21) −0.45 to 0.39  0.9051 | 0.21 (0.22)  −0.23 to 0.65 0.3387 |
| HDL (mmol/L) | | | |
| LS mean difference (SE) 95% CI  *p* value | 0.36 (0.4) −0.42 to 1.15 0.3647 | 0.22 (0.37) −0.52 to 0.96  0.5617 | 0.42 (0.44) −0.46 to 1.29 0.3476 |
| Triglycerides (mmol/L) | | | |
| LS mean difference (SE) 95% CI  *p* value | −0.17 (0.19) −0.54 to 0.20 0.3579 | 0.06 (0.14) −0.22 to 0.34  0.6789 | 0.05 (0.16) −0.26 to 0.36 0.7443 |
| Total cholesterol (mmol/L) | | | |
| LS mean difference (SE) 95% CI  *p* value | −0.4 (0.27) −0.94 to 0.13 0.1372 | 0.16 (0.25) −0.33 to 0.65  0.5281 | 0.39 (0.25) −0.11 to 0.89 0.1271 |

ALT, alanine transaminase; AST, aspartate transaminase; CI, confidence interval; EPL, essential phospholipids; GGT, gamma-glutamyl transferase; HbA1c, hemoglobin A1c; HDL, high-density lipoprotein; LDL, low-density lipoprotein; LS, least-square; SE, standard error.

**Table S13.** Change in weight from baseline to follow-up in mITT set.

| Parameter | EPL arm | Placebo arm | EPL vs Placebo |
| --- | --- | --- | --- |
| Weight (Kg) | Change from baseline | |  |
| 3 months | | | |
| n  mean (SD) | 82 −1.09 (3.846) | 83 −1.30 (3.711) | LS Mean Difference (SE): −0.09 (0.57)  95% CI: −1.22 to −1.04  *p* value: 0.8765 |
| 6 months | | | |
| n  mean (SD) | 82 −1.69 (5.188) | 82 −1.84 (8.932) | LS Mean Difference (SE): −0.40 (1.13)  95% CI: −2.63 to 1.83  *p* value: 0.7253 |
| 9 months | | | |
| n  mean (SD) | 81 −1.63 (5.644) | 82 −0.87 (9.494) | LS Mean Difference (SE): −1.31 (1.22)  95% CI: −3.72 to 1.11  *p* value: 0.2875 |

EPL, essential phospholipid; LS, least-square; mITT, modified intention-to-treat; NA, not applicable; SD, standard deviation; SE, standard error.

**Table S14.** Safety profile of EPL and placebo (PTs ≥5% in any treatment arm; safety set).

| Events | EPL arm (n = 97) n (%) | Placebo arm (n = 96) n (%) |
| --- | --- | --- |
| Number of subjects with TEAEs | 55 (56.7) | 50 (52.1) |
| Nasopharyngitis | 6 (6.2) | 6 (6.3) |
| Headache | 10 (10.3) | 8 (8.3) |
| Diarrhea | 9 (9.3) | 8 (8.3) |
| Any serious TEAE | 5 (5.2) | 3 (3.1) |
| Any study drug-related TEAE | 6 (6.2) | 5 (5.2) |
| Any TEAE leading to permanent study drug discontinuation | 2 (2.1) | 3 (3.1) |
| Any TEAE leading to permanent study discontinuation | 1 (1.0) | 1 (1.0) |
| Any TEAE leading to death | 0 | 0 |

EPL, essential phospholipid; PT, Preferred Term; TEAE, treatment-emergent adverse event

**Table S15.** Change in FIB-4 score from baseline to 6 months in mITT set.

| Parameter | EPL arm (n=82) | Placebo arm (n=83) | EPL vs Placebo |
| --- | --- | --- | --- |
| FIB-4 score | Change from baseline | |  |
| n  mean (SD) | 81 −0.069 (0.6046) | 81 0.058 (0.4147) | LS Mean Difference (SE): −0.1 (0.08)  95% CI: −0.25 to 0.06  *p* value: 0.2108 |

CI, confidence interval; EPL, essential phospholipids; FIB-4, fibrosis-4; LS, least square; mITT, modified intention-to-treat; NA, not applicable; SD, standard deviation; SE, standard error.

1. **Figures Legends**

# **Figure S1.** EXCEL study design. **^†^**Blood samples were collected to measure liver enzymes, lipid levels, glycaemic index, and HbA1c.

# **Figure S2.** Change in CAP score from baseline to 6 months by baseline subgroups

# (A) by baseline CAP score (CAP <288 dB/m and CAP ≥288 dB/m); (B) by baseline triglycerides (Triglycerides ≤1.6935 mmol/L and Triglycerides >1.6935 mmol/L); (C) by country (Germany and Poland); (D) by gender (female and male); (E) by BMI (BMI <30kg/m^2^ and BMI ≥30kg/m^2^).

# **Figure S3.** Mean (SD) scores of QoL total and subscores (CLDQ-MASLD) at baseline and follow-up visits. *Statistically significant difference for EPL versus placebo for change from baseline at month 6 (*p*<0.05)

# **Figure S4.** Percentage of patients with change in LSM score category from baseline to follow-up.

# **Figure S5.** Percentage of patients with change in CAP score category from baseline to follow-up.

1. **References**

1. EASL–EASD–EASO Clinical Practice Guidelines for the management of non-alcoholic fatty liver disease. Journal of Hepatology 2016;64:1388-1402

2. Mikolasevic I, Lukic A, Juric T, Klapan M, Madzar P, Krolo N, Kolovrat D, Jurica I, Kedmenec I, Kihas D, Ilovaca D, Erstic I, Haralovic V, Cavlina D, Dejhalla E, Erdeljac D, Vukalovic B, Skenderevic N, Milic S. Non-alcoholic fatty liver disease and transient elastography. Exploration of Medicine 2020;1:205-217

**
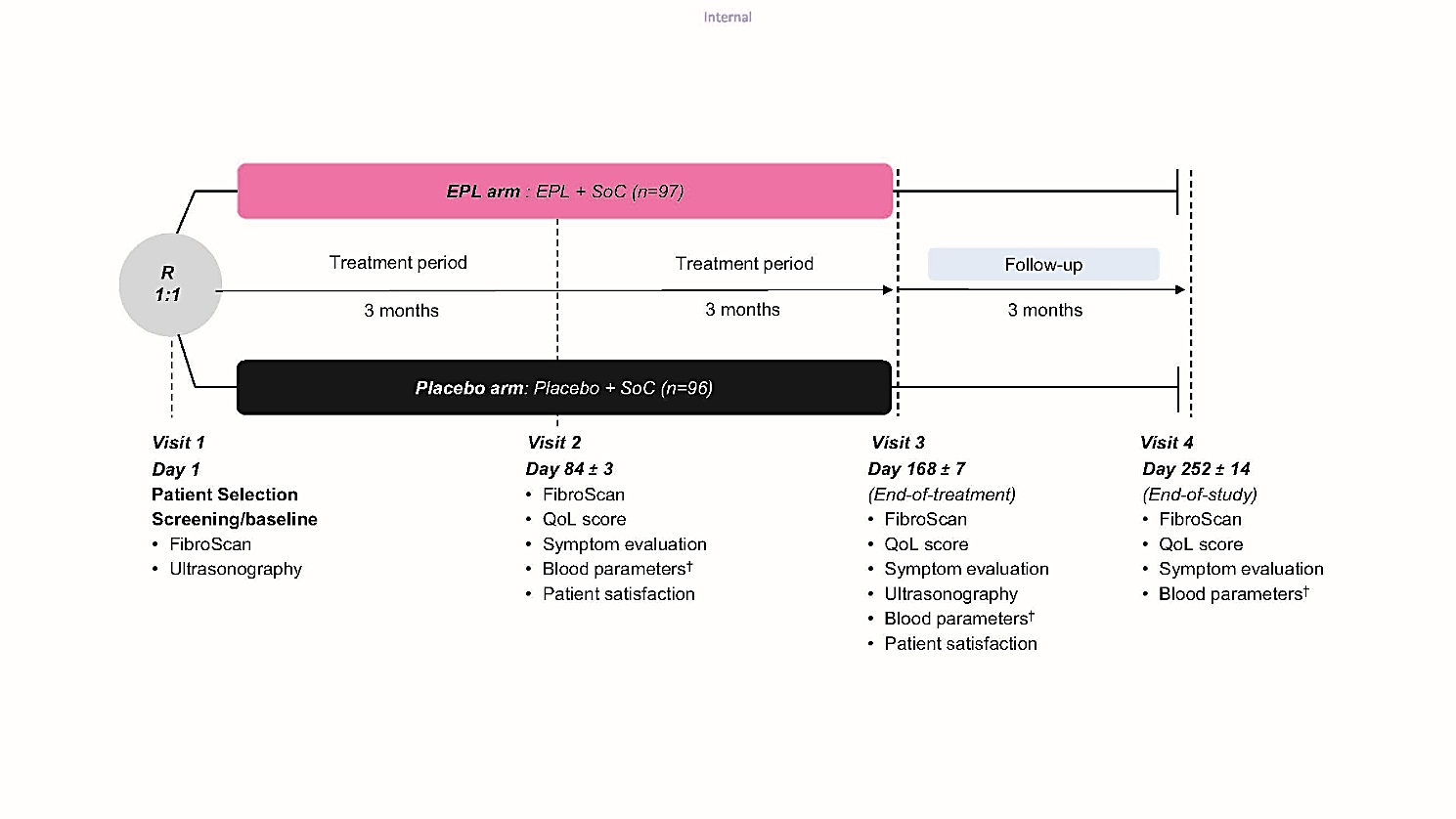
Figure S1**

**
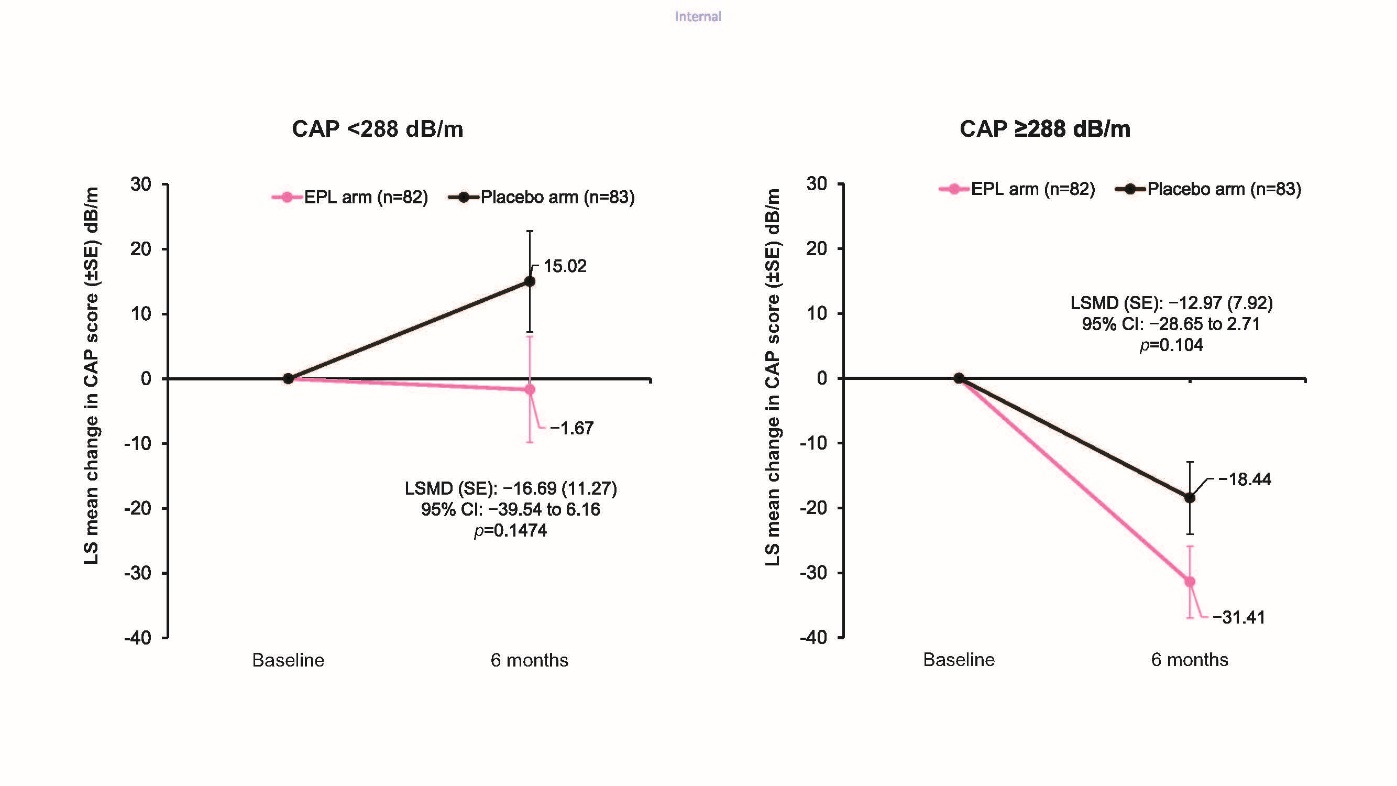
Figure S2A**

**Figure S2B**


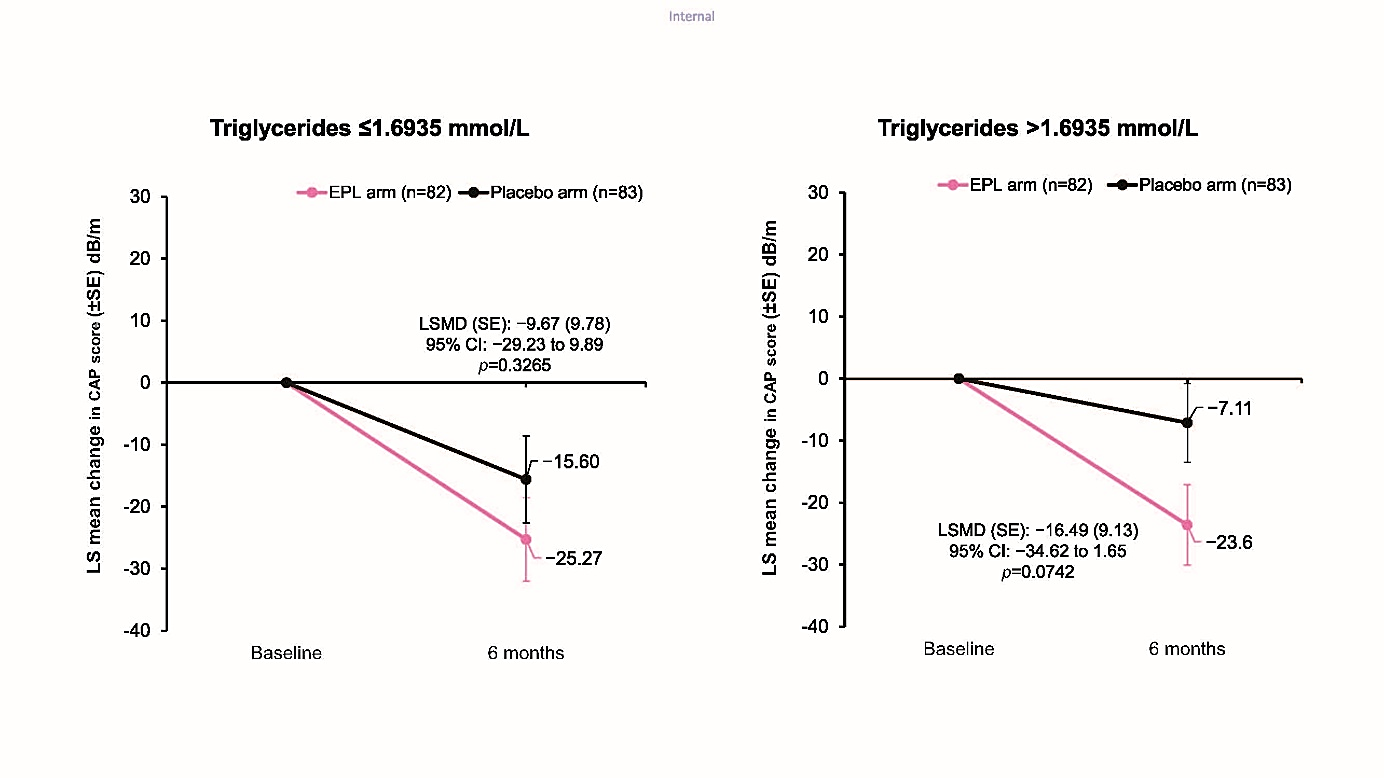


**Figure S2C**


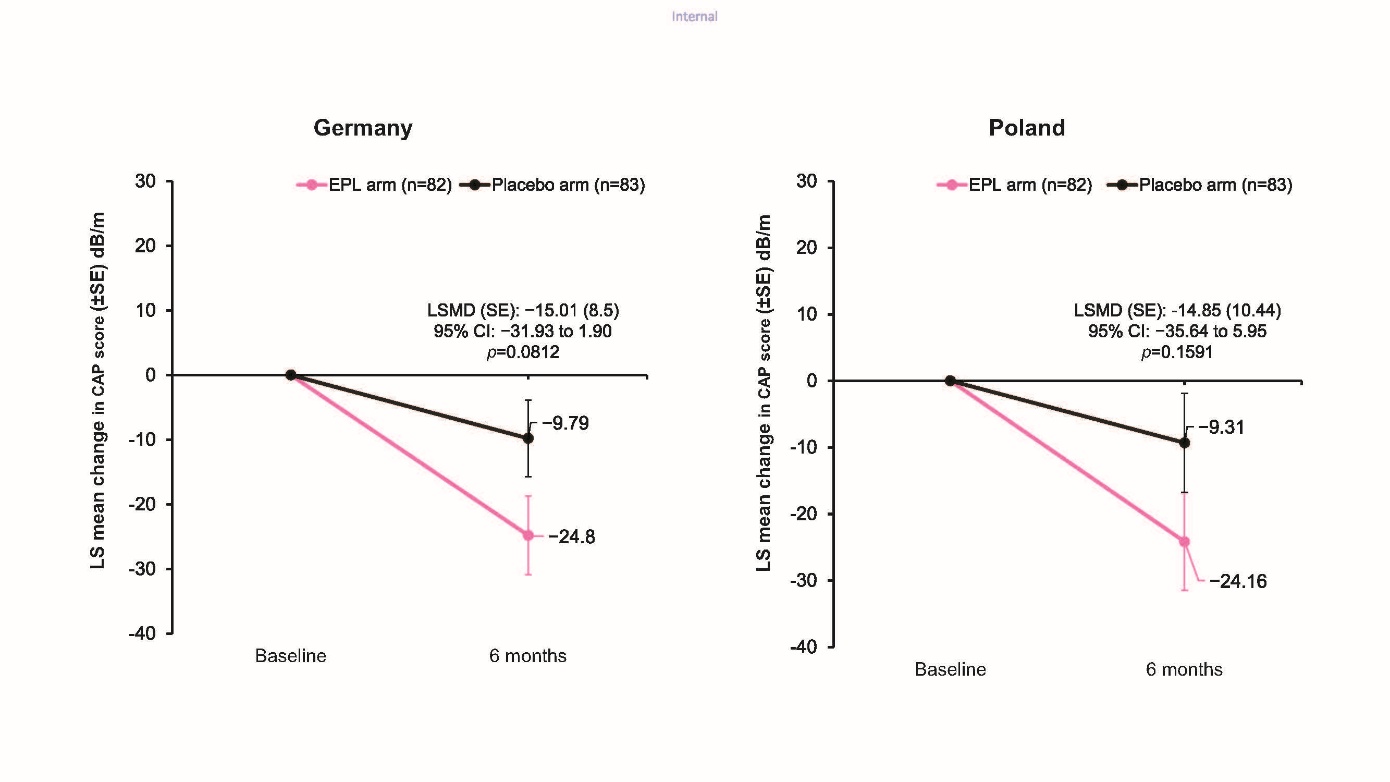


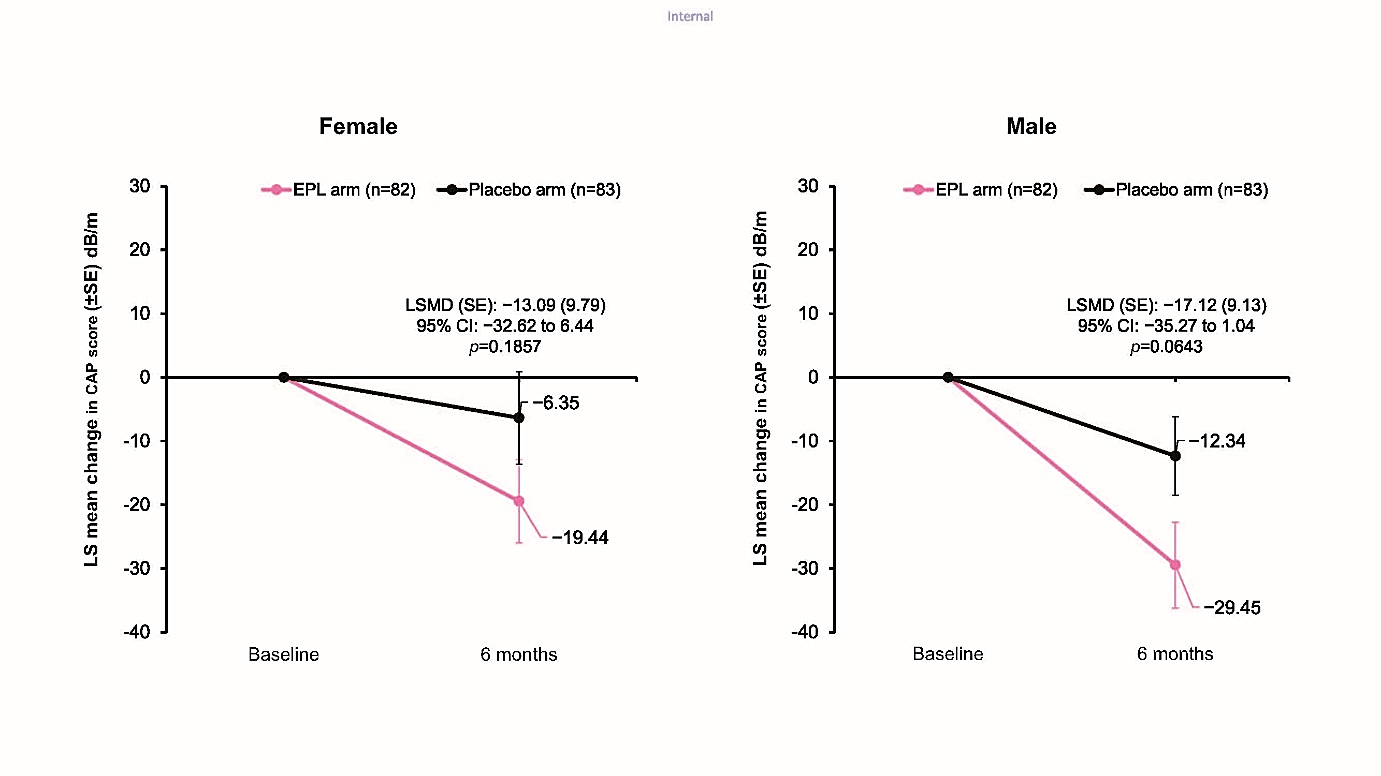
**Figure S2D**

**Figure S2E**
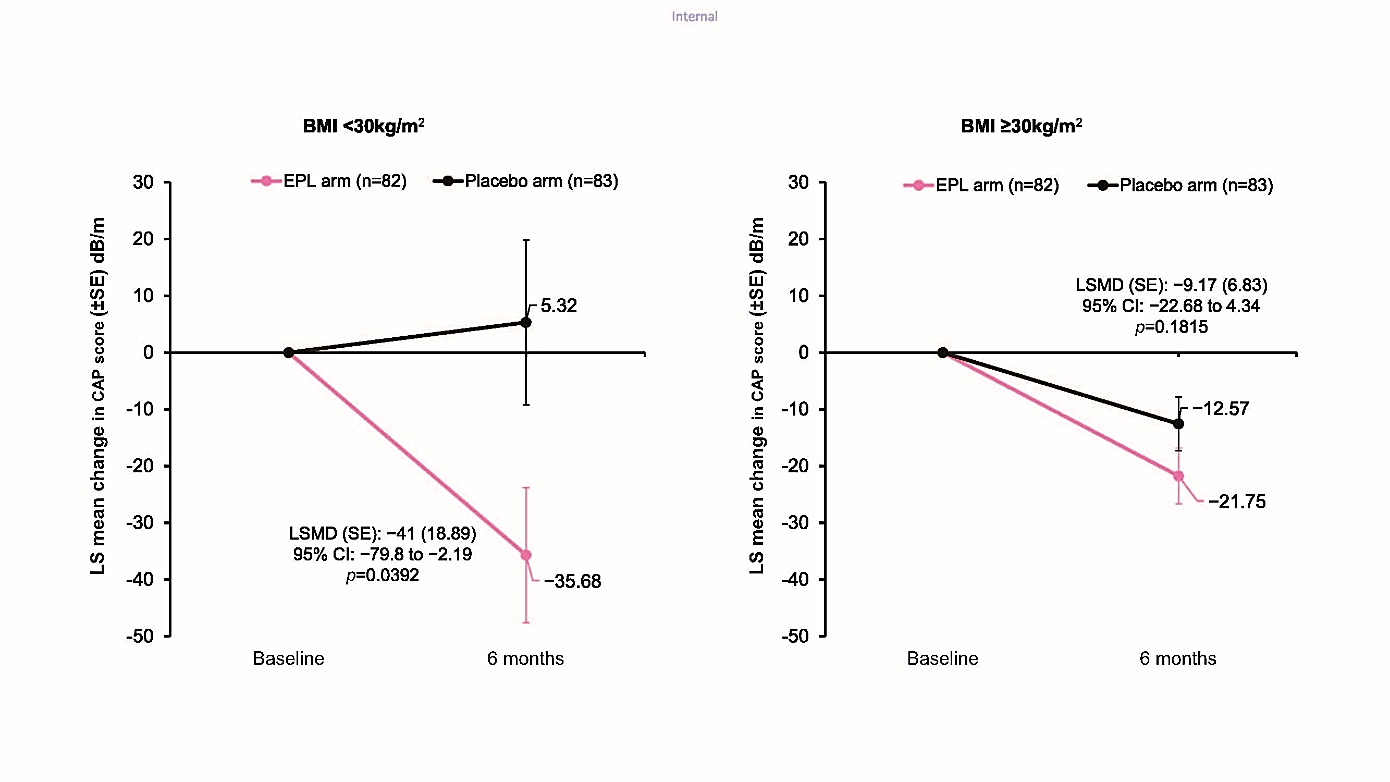


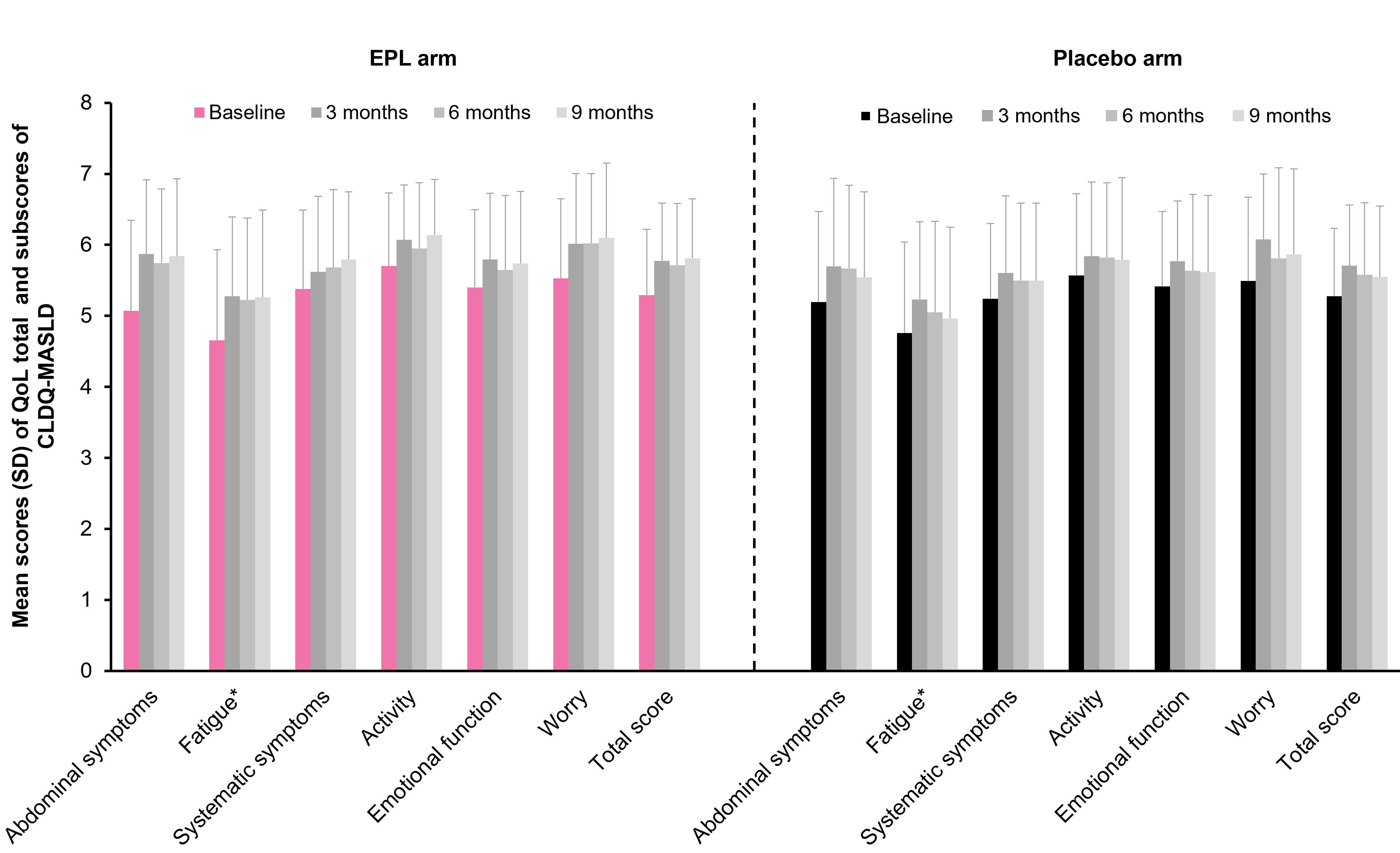
**Figure S3**


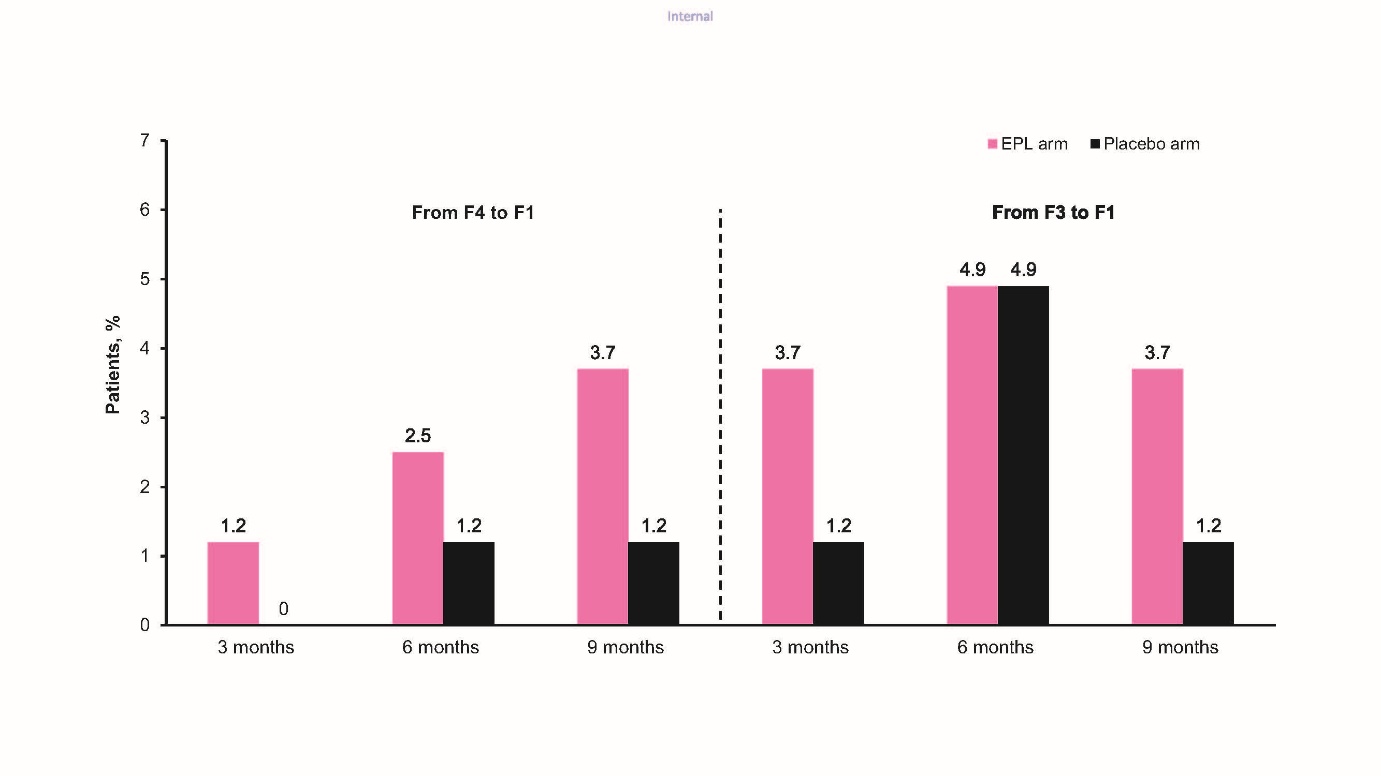
**Figure S4**

**Figure S5**
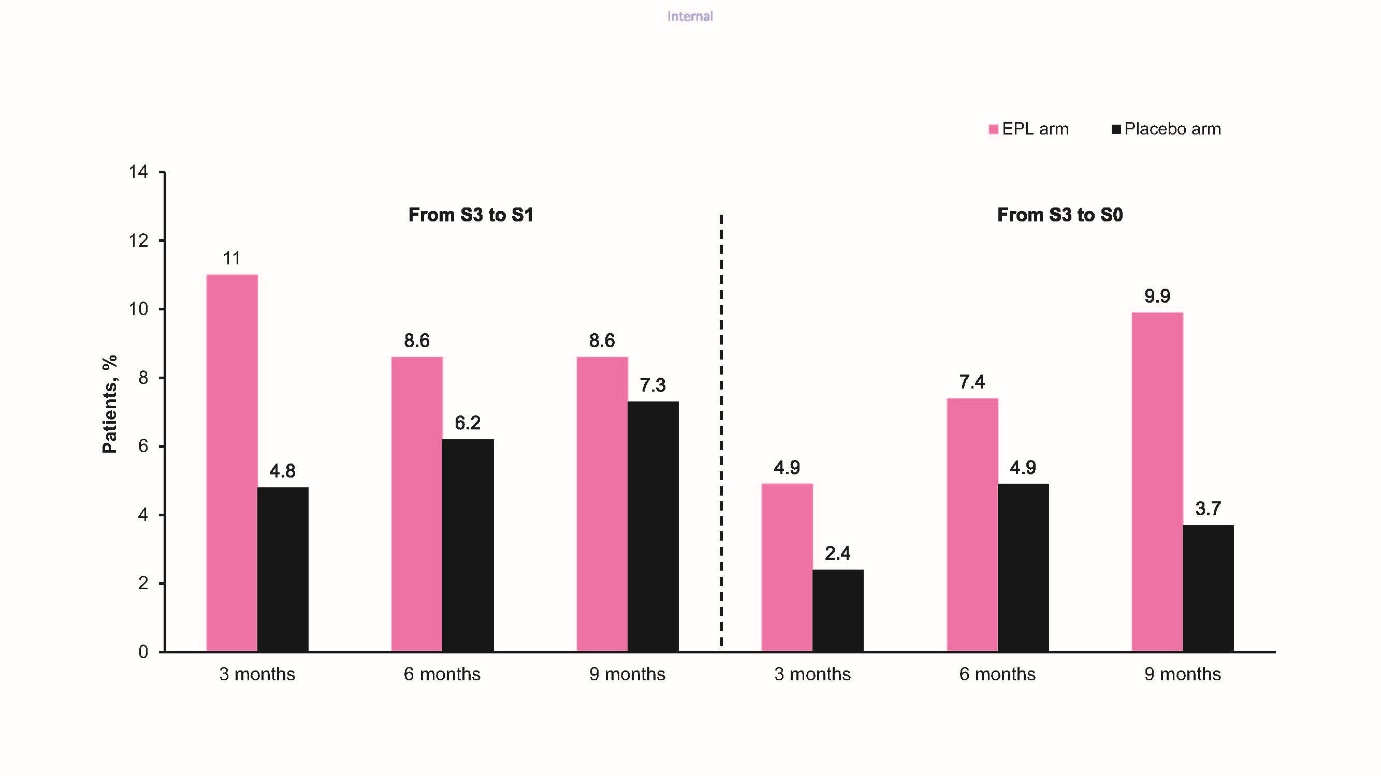

Supplement: Supplementary file 1 — Data S1: liv70601‐sup‐0001‐supinfo.docx. [file LIV-46-0-s002.docx]
